# Effect of Wet Aging on the Meat Quality of Two Cuts (*Longissimus thoracis et lumborum* and *Quadriceps femoris*) from Italian Local Goat Breeds Compared to the *Saanen* Cosmopolitan Breed

**DOI:** 10.3390/ani16010115

**Published:** 2025-12-31

**Authors:** Marica Egidio, Marika Di Paolo, Federica Capano, Sophia Alesio, Carmen Cabato, Roberta Matera, Matteo Santinello, Lucia Sepe, Raffaele Marrone

**Affiliations:** 1 Department of Veterinary Medicine and Animal Production, University of Naples Federico II, 80138 Naples, Italy; marica.egidio@unina.it (M.E.); federica.capano1997@gmail.com (F.C.); alesiosophia@outlook.it (S.A.); cabato.carmen91@gmail.com (C.C.); roberta.matera@unina.it (R.M.); matteo.santinello@unina.it (M.S.); raffaele.marrone@unina.it (R.M.); 2 CREA Research Centre for Animal Production and Aquaculture, 85051 Bella Muro, Italy; lucia.sepe@crea.gov.it

**Keywords:** electronic nose, fatty acid profile, goat meat, rheological parameters, local breeds

## Abstract

Goat meat represents a valuable source of high-quality protein and healthy lipids, although its consumption remains limited in Europe. This study aimed to evaluate the qualitative–quantitative changes in the nutritional, rheological, and sensorial characteristics of meat (*Quadriceps femoris* and *Longissimus thoracis et lumborum* muscles) from three different autochthonous goat breeds (*Garganica*, *Derivata di Siria*, and *Capra di Potenza*) and a cosmopolitan, genetically selected one (*Saanen*), reared in Basilicata (Italy), during a 7-day wet aging process. Forty kids (10 per breed) were slaughtered at 50 ± 3 days, and meat samples were vacuum-aged at 4 ± 1 °C and analyzed at 0, 3, and 7 days. Data showed that genotype was the main factor influencing meat quality, while wet aging mainly improved rheological parameters, particularly in LTL muscles. Notably, *Capra di Potenza* exhibited the most favorable fatty acid profile, with lower atherogenic (average values of 0.80 in LTL and 0.92 in QF) and thrombogenic (average values of 1.49 in LTL and 1.59 in QF) indices, whereas *Derivata di Siria* showed the greatest oxidative stability (average values of 0.060 in LTL and 0.036 in QF). Overall, local breeds of kids’ groups produced more tender and aromatic meat than *Saanen*. These findings highlight the potential of native goat breeds for premium meat production and suggest an effective post-mortem aging technique to enhance their quality, promoting the diffusion of niche products as well as biodiversity preservation.

## Simple Summary

Goat meat is a lean, highly nutritious source of high-quality protein, vitamins, minerals, and an interesting nutritional profile of fats, although its consumption remains limited in Europe. This study aimed to describe the quality characteristics of local kid goat meat (*Garganica*, *Derivata di Siria*, and *Capra di Potenza*) and cosmopolitan breed meat (*Saanen*) and to evaluate how a short wet aging period affects its nutritional and sensory traits. Meat samples were obtained from groups of goat kids belonging to different genetic types and reared under real and representative production systems, reflecting the diversity of local farming practices. Forty goat kids (10 per breed) were used, and two commercially relevant muscles were analyzed over seven days of wet aging. The results showed that meat quality reflected the combined effect of breeds, muscle type, and post-mortem aging, while wet aging mainly improved tenderness and texture. Specifically, *Capra di Potenza* exhibited the best fatty acid profile and interesting nutritional indices, whereas *Derivata di Siria* meat showed greater resistance to oxidation. Overall, local breeds produced meat that was tastier and more tender than that of the cosmopolitan breed. These findings highlight the potential of native goats to provide high-quality, sustainable meat and demonstrate how wet aging can further enhance their value, supporting the promotion of niche products and the preservation of biodiversity.

## 1. Introduction

Goat meat is widely recognized as a lean source of protein with a favorable nutritional profile [1], although its consumption remains lower compared to that of beef or pork, and it is mainly eaten during festive periods, such as Christmas and Easter [2]. However, in recent years, increasing attention to healthier diets and lean meat sources has contributed to a growing interest in this type of animal food product both in Italy and across Europe [3]. Kid goat meat is, in fact, considered a tender, easily digestible, and highly nutritious product, characterized by a pale pink color, a distinctive flavor, and a pleasant milky aroma [2]. Several studies have demonstrated that this type of meat serves as a valuable source of high-quality proteins, essential amino acids, B-group vitamins [1], and minerals, such as iron and zinc [4] and an interesting fatty acid profile characterized by an beneficial nutritional ratios [5].

Nonetheless, as highlighted by previous studies [2,3,5], the nutritional composition of kid goat meat can vary considerably. Such variability is largely influenced by both intrinsic factors, such as breed, and extrinsic factors, including environment, farming system, and diet. Notably, diet and rearing environment can affect the isotopic composition of water, fats, and proteins in animal tissues and their derived products. At the same time, genetic differences among breeds play a crucial role, influencing muscle and adipose tissue deposition, as well as the overall lipid, protein, and mineral composition of the final product [5]. Breed has also been shown to affect key quality traits from a consumer perspective, such as meat color, tenderness, and fatty acid profile [3]. Post-mortem aging is another factor that can influence goat meat quality. Studies show that aging over several days can reduce shear force (i.e., improve tenderness) and enhance desirable textural attributes [6,7] as well as induce biochemical changes in muscle tissue, including protein degradation and modifications in lipid oxidation, which contribute to flavor development and overall sensory quality [8]. Notably, different methods can be used for meat aging. Among these, the wet aging method involves the storage of fresh meat in vacuum-sealed bags, and promoting proteolytic enzyme activity led to juicier and more tender meat while reducing weight and drip losses. This technique is also preferred from an economic standpoint due to its shorter processing time and earlier market readiness of meat [9].

Therefore, considering the growing interest in kid goat meat and the scarcity of data obtained under real farming conditions, the present study aimed to provide a comprehensive evaluation of the qualitative characteristics of kid goat meat produced in the Basilicata region (Southern Italy). In particular, the study focused on two commercially relevant muscle cuts [10] obtained from animals reared under different representative production systems of the region and belonging to different genetic types, with the objective of assessing the effects of wet aging on meat quality. In this context, it should also be noted that information on the effects of aging on meat quality in sucklings (carcass weight below 7 kg) and light animals (10–13 kg carcass weight), which are widely consumed in southern Europe, remains scarce [11]. Accordingly, the *Quadriceps femoris* and *Longissimus thoracis et lumborum* muscles from three local goat breeds reared in the Basilicata region (Southern Italy), namely, *Garganica*, *Derivata di Siria*, and *Capra di Potenza*, were compared with those from a cosmopolitan, genetically selected breed (*Saanen*). The wet aging period was set at 7 days, in accordance with previous studies [12].

## 2. Materials and Methods

### 2.1. Animals’ Information

For the study, goat kids were selected from commercial farms located in the Basilicata region (Southern Italy), chosen to represent the main goat production systems currently operating in the area. Animals belonged to different genetic groups (*Garganica*, GR; *Derivata di Siria*, DS; *Capra di Potenza*, CP; and *Saanen*, SA) and were considered as representative populations of the breed. Specifically, a total of 40 male samples (10 kids per breed), aged 50 ± 3 days (54 days for the SA breed, 50 days for DS, 49 days for GR, and 48 days for CP) and with an average live weight of 13.53 ± 4.08 kg (11.42 ± 1.35 kg for GR, 13.06 ± 1.99 kg for DS, 10.25 ± 1.40 kg for CP, and 19.40 ± 3.28 kg for SA), were supplied by four commercial farms operating under real production conditions. For each breed, kids were the offspring of two different bucks and multiple does that were not siblings but only distantly related (cousins) and originated from different male lineages reared in the Basilicata region. All breeding animals have been officially registered in the respective Genealogical Books. All kids were subjected to the same experimental procedures and management conditions after selection, ensuring comparability among genetic groups.

Regarding animal feeding, pregnant local goat does (CP, GR, and DS) were reared under a semi-extensive system, whereas cosmopolitan *Saanen* (SA) goat does were managed under an intensive system, both reflecting the typical management practices adopted in the Basilicata region. For the local breeds group (CP, GR, and DS), the housing environments were maintained at an average temperature of 15–18 °C and approximately 60% relative humidity, ensured through adequate ventilation and insulation, with straw bedding kept clean and dry. From 08:00 to 16:00, does had free access to an outdoor paddock and a pasture, while kids remained indoors in the most sheltered area of the barn. Specifically, during the experimental period, does grazed in a winter pasture dominated by seasonal grass species and received breed-specific dietary supplementation. For DS, does were supplemented with 1 kg/head/day of polyphyte hay and 1 kg/head/day of a grain-based concentrate composed of 20% soybean, 25% broad bean, 50% maize, and 5% vegetable by-products to improve digestibility. For CP, does grazed in a pasture, had ad libitum access to hay, and were supplemented with 300 g/head/day of a farm-produced grain concentrate. For GR, does grazed in a pasture, had ad libitum access to hay, and received 250 g/head/day of a concentrate composed of oats and rolled grains (50% barley and 50% broad bean).

For the cosmopolitan SA group, animals were housed in a stable, with kids kept in separate stalls when the does were outside. Housing conditions were maintained at an average temperature of 20–23 °C and a relative humidity of 55–60% through adequate natural ventilation provided by permanently open high tilt-and-turn windows and ventilation chimneys, thereby limiting ammonia accumulation and excess moisture. Natural lighting was ensured by windows and open doors during the day. Animals had continuous access to clean drinking water, the milking room was separated from the housing area, and management followed a semi-intensive system, with daily access to nearby natural pastures to promote animal welfare. Regarding the feeding of does, during the experimental period, they were fed two times a day (60% morning, 20% midday, and 20% evening) with an industrial pelleted feed (18% CP for milking sheep) plus hay supplemented with mineral salt and vitamins, prepared and given through feed mixer wagon; in the study period, the diet was composed with 800 g pellet + 1200 g hay per head per day.

All kids considered in the study were suckled by their mothers overnight until 25 days of age. After this period, they were allowed to feed on polyphyte hay and a pellet concentrate composed of soybean, maize, and broad bean with an estimated intake of 10–20 g/head/day. No veterinary treatments were administered to the kids during the experimental period.

### 2.2. Experimental Design and Measurements on Carcasses

**Figure 1:**
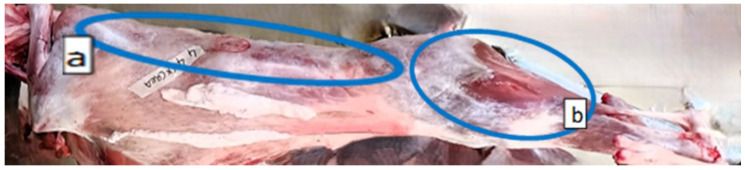
Retail muscle cuts: (**a**) *Longissimus thoracis et lumborum* (LTL) and (**b**) *Quadriceps femoris* (QF).

All kids included in the study were transported to the slaughterhouse using a vehicle authorized for the transport of live animals, in full compliance with current legislation. The distance from the farms to the slaughterhouse was 21, 40, 43, and 59 km for SA, CP, GR, and DS, respectively. Upon arrival, kids were housed in a designated lairage area and allowed to rest for at least 2 h to minimize transport-related stress. The slaughter was carried out according to Regulation (EC) No. 1099/2009 [13] on the protection of animals at the time of killing in a commercial EU-licensed slaughterhouse. Briefly, animals were handled and restrained to minimize stress, stunned using an approved method, and immediately bled, with monitoring of consciousness/unconsciousness as required by the legislation and related guidance. Following slaughter, carcasses were chilled for 24 h in a refrigerated room at 4 ± 1 °C. After chilling, from each half-carcass, two anatomical cuts, the *Longissimus thoracis et lumborum* (LTL) and the *Quadriceps femoris* (QF) muscles, were collected (Figure 1) and portioned to obtain eight LTL sub-samples (92.35 ± 66.95 g) and three QF sub-samples (213.18 ± 45.98 g). Finally, the obtained sub-samples were vacuum-packaged using vacuum bags composed of polyamide (PA) and polyethylene (PE) (20 µm of PA and 70 µm of PE in the smooth side structure; 20 µm of PA and 80 µm of PE in the embossed side structure) with an oxygen transmission rate of ≤50 cm^3^/m^2^/24 h at 1 atm (DIN 53380—23 °C 0% RH) and a water vapor transmission rate of ≤3.0 g^3^/m^2^/24 h at 1 atm (DIN 53122—23 °C 85% RH). They were then soon transported to the laboratories of the University of Naples in a cool box at approximately 5 ± 3 °C according to the UNI EN ISO 18593: 2018 [14] and placed in a cooler on a stainless-steel rack at 4 ± 1 °C. The sub-samples were analyzed at three different aging times (T0, 0 days; T1, 3 days; and T2, 7 days post-packaging). During the 7-day aging period, all analyses were performed in triplicate for each selected goat kid at each aging time using two different bags. Specifically, the physicochemical analyses (moisture, NaCl, protein and fat content, pH, water activity, fatty acid profile, and lipid oxidation) were performed using the sub-samples in one bag, while the other analyses (rheological, colorimetric, and nose–colorimetric analyses) were performed using the sub-samples in a second bag.

The dressing percentage was calculated as follows: dressing percentage (%) = (dressed carcass weight/live body weight) × 100. The pH of both sides of the carcasses at 30 min and 24 h post-slaughter, was measured manually using a portable pH meter (HI9025, Hanna Instruments, Villafranca Padovana, Italy) equipped with a meat puncture electrode, which was inserted into a small incision (2 cm depth) in the LTL between the 12th and 13th thoracic vertebrae and in QF muscles of the leg.

### 2.3. Laboratory Analyses

At the laboratory, when each package was opened, physicochemical (moisture, NaCl, protein and fat content, pH, water activity, fatty acid profile, and lipid oxidation), colorimetric and rheological analyses (color, texture profile analyses, and Warner–Bratzler shear force of meat) were carried out on raw aliquots of 3 cm thickness. In addition, for each goat breed, muscle cut (LTL and QF) and sampling time, instrumental analyses using an electronic nose were performed on both raw and cooked (heated to a core temperature of 70 ± 1 °C) aliquots to discriminate the different aroma profiles.

#### 2.3.1. Physicochemical Analyses and Fatty Acid Profile

Official methods described by the Association of Official Analytical Chemists [15] were used to determine moisture (AOAC Official Method 950.46), salt (AOAC Official Method 960.29), protein (AOAC Official Method 992.15), and fat percentage (%) (AOAC Official Method 960.39). According to Ambrosio et al. [16], pH and water activity (a_w_) measurements were carried out at room temperature (23 ± 2 °C) using a digital pH meter (Crison-Micro TT 2022, Crison Instruments, Barcelona, Spain) equipped with an insertion glass electrode and an Aqualab 4 TE device (Decagon Devices Inc., Pullman, WA, USA), respectively.

Intramuscular fat (IMF) was extracted following the procedure described by Hara and Radin [17], and the resulting samples were transesterified into fatty acid methyl esters (FAMEs) according to Di Paolo et al. [18]. The FAMEs were analyzed using gas chromatography DANI MASTER GC (DANI Corporation, Milan, Italy) equipped with a flame ionization detector (FID), a CP-Select CB for FAME capillary column (100 m × 0.25 mm i.d., 0.2 μm film thickness), and a split/splitless injector. Fatty acids were identified and quantified using pure reference standards (Supelco^®^ 37 Component FAME Mix, 47885U, Sigma-Aldrich, St. Louis, MO, USA), and the results were expressed as mg of fatty acids/100 g of meat (mg FA/100 g of meat). The proportions of saturated fatty acids (SFA_s_), monounsaturated fatty acids (MUFAs), and polyunsaturated fatty acids (PUFAs) were then determined. The PUFA/SFA ratio (P/S) and the omega6/omega3 (n-6/n-3) ratio were determined, and the nutritional index (thrombogenic index, TI, and atherogenic index, AI) and the ratio of hypo- and hypercholesterolemic acids (h/H) were also calculated as follows, according to Chen, J. & Liu, H. [19]:

$$Atherogen\;icindex\;(AI):\frac{C12:0+4\times C14:0+C16:0}{ƩMUFA+PUFA\left(n6\right)\;\mathrm{and}\;(n3)}$$

$$Thrombogenic\;index\left(TI\right):\frac{C14:0+C16:0+C18:0}{0.5\times ƩMUFA+0.5\times PUFA\left(n6\right)+\frac{n3}{n6}}$$

$$Ratio\;of\;\text{hypo-}\;and\;hypercholesterolemic\;acids\;(h/H):\;\frac{\begin{array}{c} C18:1\;cis-n9+C18:2\;cis-n6+C18:3\;n3+C20:4\;n3+\\ \;C20:5\;n3+C22:4\;n6+C22:6\;n3 \end{array}}{C12:0+C14:0+C16:0}$$

Moreover, according to Migdal et al. [20], the meat softness index and the nutritional value of lipids were calculated as follows:

$$Meat\;softness\;index\;(SI):\frac{C16:1+C18:1}{C16:0+C18:0}$$

$$Nutritional\;value\;of\;lipids\;(NV):\;\;\frac{C12:0+C14:0+C16:0}{C18:1c9+C18:2n6}$$

#### 2.3.2. Lipid Oxidation (TBAR_s_)

The lipid oxidation (TBAR_s_) was evaluated by quantifying the thiobarbituric acid substances following a modified extraction method described by Di Paolo et al. [18]. The results were expressed as mg of malondialdehyde (MDA), a secondary lipid oxidation product, per kg of meat according to the following formula:

TBAR value (mg/kg) = A_532_ * × 7.8

* A_532_ = wavelength absorbance at 532 nm.

#### 2.3.3. Colorimetric and Rheological Analyses

For each goat kid, color measurements of raw meat were performed on LTL and QF muscles at three different surface points for each aging time (T0, T1, T2) using a Konica Minolta CR-300 colorimeter (Minolta, Osaka, Japan) with an 8 mm diameter measuring aperture, calibrated against a white standard plate. The operative conditions were Illuminant D65 and a 10° standard observer. The lightness (L*), redness (a*), and yellowness (b*) parameters were recorded by considering the SCI (specular component included) measurement, while chroma [(a*^2^ + b*^2^)^1/2^] and hue angle (Tan^−1^ b*/a*) were calculated as described by Marrone et al. [21] and Ambrosio et al. [16].

The texture profile analysis (TPA test) and the Warner–Bratzler shear force (WBSF) tests were assessed on raw QF and LTL muscles at all sampling times (T0, T1, and T2) using an EZ-Test texturometer Shimadzu (Shimadzu Corporation, Kyoto, Japan) as reported by Ambrosio et al. [16] and Marrone et al. [21], respectively. Notably, the TPA test provided the following parameters: adhesiveness, hardness, springiness, gumminess, chewiness, and resilience, while the WBSF test measured the shear force (N) as an indicator of meat toughness [22]. The mean values of the cuts were used for the statistical analysis.

#### 2.3.4. E-Nose Analysis

The aroma characteristics of each goat breed were evaluated on raw and cooked meat samples using the PEN3.5 portable electronic nose (E-nose PEN3.5, Airsense Analytics GmbH, Schwerin, Germany) with a headspace aspiration method. This system, equipped with ten different metal oxide sensors, each selective and sensible for a specific molecular group, as shown by Xing et al. [23], was used to characterize breed-specific aroma traits and to monitor changes in volatile compounds in the LTL and QF muscles during aging times (T0, T1, and T2).

Briefly, a portion of LTL and QF raw muscle (2.5 cm × 2.5 cm) from each goat kid was placed in a 50 mL glass vial fitted with a septum cap and equilibrated for 30 min at room temperature (25 ± 1 °C). For the cooked samples, a portion of LTL and QF raw muscle (2.5 cm × 2.5 cm) was thermally treated in an electric furnace at 180 ± 2 °C until an internal muscle temperature of 70 ± 1 °C, as measured with a digital probe thermometer. After cooking, each sample was placed in the 50 mL glass vial, cooled for 30 min at room temperature (25 ± 1 °C), and analyzed. Before and after each measurement, a flushing phase (cleaning time) was performed using an air washing system, in which room air was passed through an activated carbon filter to remove interfering gases and purge residual volatiles from the sensors, thereby standardizing them to the analytical environment and minimizing measurement error. Each vial was then connected to the PEN3.5 system through a sterile needle (replaced for each sample) [24].

The real-time sensor responses were acquired at 1 s intervals, resulting in 1200 corresponding data per raw sample (120 response values × 10 sensors) and 2000 per cooked sample (200 response values × 10 sensors). Overall, the chosen E-nose operating parameters were as follows: sensor cleaning time, 300 s; auto-zero time, 10 s; sample preparation time, 5 s; sample measurement interval, 1 s; internal flow, 600 mL/min; injection flow, 600 mL/min; and measurement time, 120 s for raw samples and 200 s for cooked samples.

### 2.4. Statistical Analysis

Statistical analyses were performed using the SPSS program, version 30 (IBM Analytics, Armonk, NY, USA), and WinMuster software v.1.6 for electronic nose systems. Physicochemical and rheological data, expressed as mean ± standard error, were statistically analyzed using a multivariate general linear model (GLM), considering aging time (T0, T1, T2), breed group (GA, DS, CP, SA), and muscle (LTL and QF) as fixed factors. Normality and homoscedasticity were verified by Shapiro–Wilk and Levene’s tests, respectively. Helmert contrasts were applied for aging time, breed, and muscle effects, with Bonferroni correction used for multiple comparisons. Tukey’s test was used to calculate *p*-values (*p* < 0.05 and *p* < 0.01). Each animal belonged to a single breed, and the breed was considered the main grouping factor in experimental design, with animals evaluated within each breed.

For the sensorial E-nose data, the optimized sensors’ response values obtained from WinMuster software were subjected to principal component analysis (PCA) using the SPSS program to investigate breed-specific as well as muscle cut differences in aromatic profiles and their evolution over aging. In this regard, two separate PCAs were performed: one for the raw meat samples, including both QF and LTL muscles of all breeds and sampling times, and one for the cooked samples, analyzed under the same conditions.

## 3. Results and Discussion

### 3.1. Slaughtering Goat Kids’ Carcasses Parameters

As shown in Table 1, data revealed that CP and GR kids exhibited the most favorable aptitude for meat production, as they had higher slaughter yields (%) compared to the other two, despite the lower live weights (kg) and age (Section 2.1). The obtained results can plausibly be attributed to a greater aptitude of the DS and SA breeds for milk production, as reported by other authors [25]. Similar findings regarding GR and DS goat breeds have been described by Colonna et al. [3] and by Ibrahim et al. [26] for the SA breed. By contrast, information regarding CP is still very limited, as this autochthonous breed is currently at risk of extinction and is reared in very small numbers.

Regarding pH measurements, a significant (*p* < 0.01) pronounced decline was observed in both LTL and QF muscles after 24 h post-slaughter, with values decreasing from approximately 7.4–6.8 to about 6.3–5.5 (Table 1). This reduction is consistent with the normal post-mortem glycolytic changes occurring in muscle tissue, which are crucial for both limiting bacterial growth [27] and maintaining meat stability and quality [18]. Comparing the two muscle cuts, a relevant observation is that QF generally exhibited higher pH values than LTL, except for SA kids. This behavior is likely linked to the intrinsic differences in muscle fiber composition, since variations in the proportion of red (oxidative) and white (glycolytic) fibers are known to modulate acidification kinetics and metabolic patterns both ant- and post-mortem [28]. Indeed, muscles with a higher proportion of slow-twitch red fibers, characterized by abundant oxidative enzymes and limited glycogen reserves, tend to reach higher ultimate pH values compared with muscles predominantly composed of fast-twitch white fibers, which typically exhibit lower ultimate pH [18]. These results agree with those of Santos et al. [28], Bonvillani et al. [29], and Colonna et al. [3], who also conducted studies on goat kid meat. Breed-group differences were also evident, with GR exhibiting the lowest pH values at 24 h and experiencing the most pronounced decline in both muscles, followed by DS. These observations align with previous findings reported by Colonna et al. [3] and Rotondi et al. [30], suggesting that breed-specific variations in muscle metabolism may influence post-mortem acidification patterns. In contrast, the other two group breeds (CP and SA) showed a less marked, yet statistically significant (*p* < 0.01), reduction in pH compared with those reported in previous studies [31,32], as their values at 24 h remained above 6.0, which is considered a critical threshold for meat quality [31]. However, high ultimate pH values for goat muscles are prevalent in the literature [33,34], suggesting that goats generally may be highly prone to different forms of stress [1,35].

### 3.2. Meat Quality of Goat Kids

Table 2 shows the pH and water activity (a_w_) values measured on the LTL and QF muscle cuts of goat kids from the four considered breeds during the aging period. Overall, pH values remained relatively stable in both muscle cuts, ranging mostly between 5.5 and 5.7. Notable exceptions were observed in SA, which reached 5.9 in the LTL muscle, and in CP, where both LTL and QF attained values close to the threshold limit for acceptable goat meat quality [31] on day 7 (T2). Despite these variations, no statistically significant differences were found between the four breeds (Table S1), and excluding CP, final pH values on day 7 ranged between 5.72 and 5.92, falling within the interval associated with normal glycolytic activity and good meat quality [31]. Regarding a_w_, the LTL and QF muscles showed stable values throughout the aging period, with a significant decrease observed only at T2 in the QF muscle of GR kids (from 0.985 to 0.979; *p* < 0.05). Our data are in accordance with those reported by Ali et al. [36] on *Longissimus lumborum* (LL) and *Biceps femoris* (BF) muscles from Korean native black goat meat and Gürbüz, Ü. et al. [37] on lamb loins meat.

The chemical composition of LTL and QF muscle cuts from goat kids belonging to the GR, DS, CP, and SA group breeds over the wet aging period is presented in Table 3. Overall, regardless of aging time, differences were mainly associated with the considered anatomical muscle cuts and goat breeds. Notably, the chemical composition of goat kid meat was primarily influenced by breed, with the SA kids group distinguished by higher protein and salt percentage, while CP and GR were distinguished by greater fat content. Muscle type played a secondary role, affecting mainly fat distribution, while the aging period exerted only limited effects on chemical parameters, with minor reductions in fat content and small, inconsistent shifts in moisture, protein, and salt over time. These findings indicate that short-term post-mortem storage (up to 7 days) did not substantially alter the proximate composition of kid meat, and that inherent breed differences were more relevant than aging or muscle type in determining the chemical profile.

Specifically, the moisture content (%) remained within a narrow range (74–76%) across all breeds, muscle cuts, and aging times (Table 4), indicating that wet aging exerted only a limited effect on the overall moisture balance of the meat [38]. Statistically significant differences (*p* < 0.05) were only observed for the LTL muscle between GR with both CP and SA at T0, as well as within the GR kids between T0 and T1 aging time.

Regarding fat percentage (Table 3), as expected for young animals [39], values were low in all groups of kids (2–3%) but showed marked variability. At T0, the local breeds group, notably GR and CP, displayed higher fat content, particularly in LTL (3.16% in GR and 3.31% in CP), while DS and SA revealed lower levels (2.31% in DS and 2.39% in SA). Aging was generally associated with a slight decline in fat percentage, which was most evident in LTL of GR (*p* < 0.05) and was probably associated with the oxidative and lipolytic processes that occurred during the storage period [35]. Within the breeds group, LTL consistently contained more fat than QF, highlighting a muscle-dependent deposition pattern [40]. Also, for this parameter, statistically significant differences (*p* < 0.01) were observed in the LTL muscle between GR and DS at time T0 and within the GR kids (*p* < 0.05) at time T0 with both T1 and T2.

As for protein content (Table 3), levels were relatively stable, but differences among breeds were observed. SA was characterized by the highest protein contents (19–20%), followed by CP, DS, and GR. During the aging time, some breeds (i.e., GR and DS) showed slight increases in protein values from T0 to T1, after which the values stabilized. Muscle-related differences were less evident than breed effects, although the LTL muscle of SA consistently showed slightly higher protein values than QF at the beginning of aging. Statistically significant differences (*p* < 0.01 and *p* < 0.05) were observed in most cases between the local breeds and the selected one (*Saanen*) for both LTL and QF muscles.

Finally, with respect to salt concentration (NaCl) (Table 3), values were observed to range between 0.5% and 0.9%. SA exhibited the highest content (0.89 for QF and 0.85% for LTL), especially at T0, while GR showed the lowest (0.57% in LTL and 0.55% in QF). These differences are probably linked to the genotype, as evidenced by the significant breed-related effect (*p* < 0.01) observed on this chemical parameter. No significant differences were found between samples. The obtained data are in accordance with those previously reported by Shi et al. [38] on aging beef; Kawęcka et al. [41] on male kids meat from the Polish Carpathian native goat breed slaughtered at different ages; Martinez et al. [39] on *Longissimus dorsi* (LD), *Biceps femoris* (BF), *Semimembranosus* (SM), and *Semitendinosus* (ST) muscles of goats; and Kapase et al. [42], who examined the post-mortem aging characteristics of hot-boned sheep meat.

### 3.3. Fatty Acid Profile of Goat Kids’ Meat

**Figure 2:**
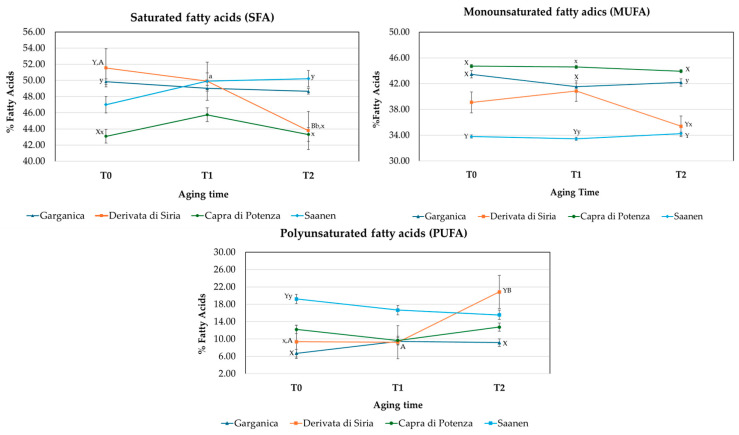
Changes in the content of saturated fatty acids (SFAs), monounsaturated fatty acids (MUFAs), and polyunsaturated fatty acids (PUFAs) during aging time (T0, 0 days; T1, 3 days; and T2, 7 days) in the *Longissimus thoracis et lumborum* muscle cut from the four groups of kids (GR, *Garganica*; DS, *Derivata di Siria*; CP, *Capra di Potenza*; SA, *Saanen*). Different lowercase (a,b = *p* < 0.05) or uppercase (A, B = *p* < 0.01) letters indicate significant differences among aging times within the same breed. Different lowercase (x, y = *p* < 0.05) or uppercase letters (X, Y = *p* < 0.01) indicate significant differences among breeds for the same aging time.

**Figure 3:**
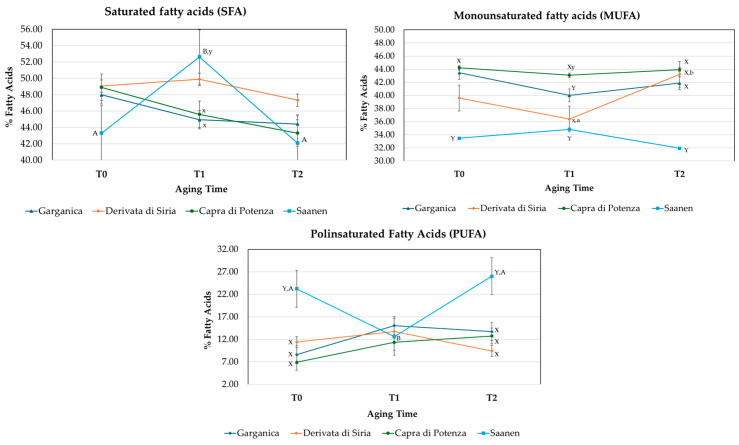
Changes in the content of saturated fatty acids (SFAs), monounsaturated fatty acids (MUFAs), and polyunsaturated fatty acids (PUFAs) during aging time (T0, 0 days; T1, 3 days; and T2, 7 days) in the *Quadriceps femoris* muscle cut from the four groups of kids (GR, *Garganica*; DS, *Derivata di Siria*; CP, *Capra di Potenza*; SA, *Saanen*). Different lowercase (a, b = *p* < 0.05) or uppercase (A,B = *p* < 0.01) letters indicate significant differences among aging times within the same breed. Different lowercase (x, y = *p* < 0.05) or uppercase letters (X, Y = *p* < 0.01) indicate significant differences among breeds for the same aging time.

The fatty acid composition in LTL and QF muscles from different goat breeds changed significantly among groups (GR, DS, CP, SA) and aging times (Tables S1 and S2; Supplementary Materials). Overall, breed exerted a stronger influence (*p* < 0.001) than aging time on the LTL muscle fatty acid profile (Figure 2). Conversely, the QF muscle showed greater temporal variability (*p* < 0.001), indicating a higher sensitivity to aging processes (Figure 3). These results support the hypothesis that anatomical and metabolic characteristics of muscle type play a key role in modulating oxidative stability during post-mortem aging [43]. Goat breed significantly affected (*p* < 0.001; *p* < 0.01) the proportions of individual saturated fatty acids (SFAs), except for stearic acid (C18:0), which was in agreement with Yalcintan et al. [44] and Colonna et al. [3]. In this context, CP kids consistently exhibited the lowest proportion of SFAs, mainly due to the reduced levels of lauric (C12:0), myristic (C14:0), and palmitic (C16:0) acids, resulting in a more favorable lipid profile [45]. Accordingly, CP and GR displayed higher proportions of monounsaturated fatty acids (MUFAs) compared to SA and DS kids’ meat, particularly oleic acid (C18:1 cis-9), which is associated with beneficial health effects [46]. These findings are consistent with previous observations in *Carpathian* and *Saanen* goats [20]. Regarding polyunsaturated fatty acids (PUFAs), especially linoleic (C18:2 n-6) and arachidonic acid (C20:4 n-6), intramuscular fat from SA kids showed higher proportions of these fatty acids compared with the local breeds group, confirming earlier reports in *Saanen* goats [20,44]. In contrast, the proportion of PUFA n-3 fatty acids (EPA and DHA) was higher in CP and GR, confirming breed-related variability reported by Liotta et al. [47] and Ivanović et al. [48]. Notably, GR showed a marked rise in EPA and DHA after 3 days of aging (*p* < 0.01), suggesting higher desaturase activity and potentially improved nutritional quality of its intramuscular fat. Similar results have been reported by Sikora and Borys [49,50], who demonstrated that desaturase activity and unsaturated fatty acids in goat muscles are influenced by animal age and muscle metabolic rate. These findings align with other studies showing that lipid profile and Δ^9^-desaturase activity vary with muscle type, physiological maturity, and metabolic characteristics [51,52]. Consequently, DS and CP exhibited the lowest n-6/n-3 ratios, indicating a more favorable nutritional quality of their meat compared with the other breeds.

Meat from DS kids exhibited a higher concentration of conjugated linoleic acid (CLA) isomers than the other breeds (*p* < 0.05). CLA values in ruminant meat and milk are affected by factors such as genotype, age, and diet [53]. Although CLA represents a minor portion of total fatty acids, it plays a key role in human health, showing beneficial effects against cancer, atherosclerosis, obesity, osteoporosis, and inflammatory processes [54,55]. In this study, CLA values agree with those reported by Peña et al. [56] in Criollo Cordobes goats and in Anglo-Nubian kids. It should also be noted that slaughter age significantly affects the fatty acid profile of goat meat. In our study, goats were slaughtered at 50 ± 3 days of age, while Horoszewicz and Pieniak-Lendzion [57] observed more favorable fatty acid parameters in White Improved breed goat kids slaughtered at 150 days compared to those slaughtered at 90 days.

Moreover, the thrombogenic index (TI) and atherogenic index (AI) were calculated to assess the nutritional quality of lipids in both muscles, as these indices provide valuable information on the potential effects of dietary fatty acids on cardiovascular health. Elevated TI and AI values are generally associated with an increased risk of thrombosis and the development of atherosclerosis [20]. Furthermore, the nutritional value of lipids (NV) was also evaluated, since higher AI, TI, and NV values are typically indicative of lower meat nutritional quality [58].

The nutritional indices of intramuscular fat showed clear differences among breed groups and aging times in both muscles. In LTL (Table 4), breed exerted a significant effect on most parameters, particularly on the h/H, SI, and NV indices (*p* < 0.001). The CP kids consistently displayed the most favorable lipid profile, characterized by a lower atherogenic and thrombogenic index (AI and TI) and a higher h/H ratio, suggesting a healthier fatty acid composition [19].

In the QF muscle (Table 5), temporal variations were more pronounced, particularly in the SA, where significant fluctuations were observed in n-6, TI, and NV indices (*p* < 0.01). Despite these variations, CP again maintained favorable nutritional ratios, while GR and DS showed more stable but less advantageous profiles. Overall, the results indicate breed-dependent differences in lipid quality and suggest that both muscle type and post-mortem aging influence the nutritional value of goat meat [43].

Fatty acids are also key determinants of meat sensory attributes, particularly tenderness, palatability, and aroma [20]. Notably, the ratio of unsaturated fatty acids (C16:1 + C18:1) to saturated fatty acids (C16:0 + C18:0) serves as an important indicator of meat tenderness, referred to as the meat softness index (SI). In this regard, the highest SI values were observed in CP kid meat for both the LTL (from 1.18 to 1.21) and QF (from 1.13 to 1.21) muscles, further confirming that it has a nutritional profile more favorable to consumer preferences compared to those of the other goat breeds.

### 3.4. Lipid Oxidation (TBAR Test) of Goat Kids’ Meat

The measurement of thiobarbituric acid reactive substances (TBARs) is widely applied to evaluate the extent of lipid deterioration in muscle foods, as these compounds react with malondialdehyde (MDA), which is a secondary lipid oxidation product used as one of the most peroxidation biomarkers [59]. This process generates free radicals, which promote the meat pigment oxidation, leading to the development of rancid odors as well as off-flavors during storage that are carefully monitored, as they negatively impact the overall quality of the final product [60]. The TBAR values obtained in this study (Table 5) reflect the lipid oxidation in goat meat from the four different groups of kids considered (GR, DS, CP, and SA) during the chosen standardized wet aging period. As expected, all breeds showed an increase of TBAR values over time since during the oxidation of unsaturated fatty acids, degradation products of lipid hydroperoxides and peroxides gradually accumulate [61]. This increase can be partly explained by post-mortem enzymatic activity, as endogenous lipases remain active during refrigerated storage and wet aging, promoting lipid hydrolysis and the release of free fatty acids, which are more susceptible to oxidative reactions [62]. Consequently, TBAR values appeared to be storage time-dependent, confirming that lipid oxidation intensified as the wet aging period progressed, as was previously observed by Wang et al. [63] for fish product, Forte et al. [64] for goat meat, and Di Paolo et al. [18] for bovine meat. Specifically, this oxidative analysis revealed higher TBAR values in SA, particularly for the LTL muscle, ranging from 0.2 at T0 to 0.4 mg MDA/kg at T2, with a statistically significant increase (*p* < 0.01) between the two aging times (Table 6) and lower values in the other three local breeds groups (CP, GR and DS), none of which exceeded 0.2 mg MDA/kg for both muscles at all aging times. The higher TBAR values observed in the SA kids, followed by the GR, may reflect breed-related differences in lipid metabolism, including a higher degree of fatty acid unsaturation and, in particular, a greater polyunsaturated fatty acid content. This condition, potentially associated with variations in the activity of enzymes, such as Δ^9^-desaturase, can modulate the intramuscular fatty acid profile and increase the susceptibility of meat lipids to oxidative processes, thereby affecting oxidative stability [51,52]. Despite these variations, a statistically significant difference from SA was only observed in local kids breeds at the end of the storage (7 d). DS was the exception among the three, showing the lowest TBAR values (0.03–0.07 mg MDA/kg) in both LTL and QF muscle cuts, with already significantly lower levels than SA at T0 (*p* < 0.05), which became even more pronounced (*p* < 0.01) at later time points. It is important to underline that all values remained below 2.0 mg MDA/kg, which is considered the limiting point for the onset of sensory rancidity from where rancid flavor overpowers meat flavor [65]. Differences in TBAR_s_ were also observed between muscle cuts of the same breed, although they were not statistically significant. Overall, QF showed lower values compared to LTL muscles, likely due to its minor fat content [66], which is in agreement with the proximate composition data reported in Table 3. Indeed, the meat lipid oxidation degree, in addition to being influenced by the aging process duration, also depends on factors such as the intramuscular fat (IMF) content and the degree of fatty acids unsaturation [66]. Overall, the effects of wet aging on lipid oxidation should, therefore, be interpreted not only as a storage time-dependent phenomenon but also as the result of complex biochemical processes involving enzymatic activity and breed-specific metabolic traits. Similar trends and results in goat meat were also reported by Sabow et al. [67], Adeyemi et al. [68], and Forte et al. [64].

### 3.5. Color of Goat Kids’ Meat

Meat color is a key determinant of perceived quality, as it represents the first visual attribute assessed by consumers. Notably, a brilliant red appearance is often regarded as a marker of superior quality and freshness [69]. Deviations from this characteristic color typically result in product rejection and consequent economic losses [70]. Beyond its aesthetic and commercial relevance, color also holds physiological and biochemical significance, as it provides indirect information on key parameters such as oxygen availability, myoglobin content, and muscle pH. Table S3 shows the changes in color properties, including lightness (L*), redness (a*), and yellowness (b*) values of goat LTL and QF muscles from the four breeds group (GR, DS, CP, and SA) during the aging period. Further statistical information, as well as hue and C* values, are reported in the Supplementary Materials (Table S3). As was observed, color parameters (L*, a*, b*) varied among breeds, muscle types, and aging times, although most differences were not statistically significant. For lightness (L*), whose values are generally influenced by numerous complex factors (e.g., muscle structure (especially fat deposition), pigmentation, ultimate pH, lighting conditions) and their potential interactions [71], GR maintained stable values across aging, with no significant differences between LTL and QF (Table S3). In DS, a slight increase at T1 was observed in QF, but values returned to baseline by T2, while LTL remained stable. CP showed a significant decrease (*p* < 0.01) in LTL from T0 to T2, probably related to the lipid oxidation processes that occur during the aging period, promoting metmyoglobin formation [72]. In the same way, in the QF muscle, the L* values showed a significant decrease (*p* < 0.05) at T0 compared to LTL, further confirming that higher pH levels result in darker meat [73]. Finally, regarding SA kids, both LTL and QF anatomical cuts exhibited significantly lower L* values (Table S3) compared with those of the other three at all aging times, highlighting the overall darker appearance of this meat. No significant changes were observed in the same breed during the wet aging period.

Regarding redness (a*), primarily determined by myoglobin content and its oxidation state [74], GR and CP displayed stable values over time, highlighting the role of wet aging in preserving them from discoloration, maintaining color stability. In contrast, DS exhibited a statistically significant increase (*p* < 0.05) in LTL from T0 to T1 (Table S3). Regarding SA, both LTL and QF muscles presented the highest a* values throughout the aging period, significantly exceeding those of GR (*p* < 0.05 for LTL muscle; *p* < 0.01 for QF) and DS (*p* < 0.05 for LTL muscle) at T0 and T1 (*p* < 0.01 for DS QF and for both muscles in GR). The increase in redness, observed in most cases at the end of the aging period, may reflect a higher pigment concentration as well as the effect of blooming, a dynamic process primarily caused by the formation of a thin layer of oxymyoglobin on the meat surface upon opening the vacuum package [75,76].

For yellowness (b*), which reflects the presence of meat pigment cells and fat-related compounds [77], GR and CP remained stable across time points and muscle cuts. Only a statistical difference (*p* < 0.01) was observed at T1 for CP between QF and LTL, with a higher b* value in the QF muscle. DS showed a marked decrease in LTL at T1 (from 11.74 at T0 to 8.63 at T1) that persisted at T2 (8.61), while QF values declined moderately (from 10.82 to 9.02). Regarding SA, lower but not statistically significant b* values in LTL compared to QF (Table S3) were observed, with slight increases over time, probably due to the meat pigment cell degradation or myoglobin oxidation, processes known to enhance yellowness during storage [77]. Moreover, SA exhibited lower b* values compared to the other three kids’ groups, showing significant differences (*p* < 0.01) compared to DS at T0 for LTL muscles and then CP at T1 for QF (Table S3). Overall, the b* axis in the local breeds groups ranged between 9 and 11, whereas that of SA ranged from 5 to 8 at T0 (Table S3). Similar a* and b* values were observed by Jose and McGilchrist [75] on beef samples, and similar trends were reported by Smeti et al. [78] on beef meat and Muhammad and Sabow [79] on goat muscles.

Finally, regarding chroma (C*) and hue angle (h°), lower C* values were detected in SA samples, although no statistically significant differences were observed (Table S3). The SA kids likewise showed significantly higher hue values compared with those of the other three groups. Previous studies [76,80] have reported that low chroma values combined with high hue values are indicative of a meat with a less saturated and intense color, tending to appear visually less bright, dull, and grayish, with a darker, gloomier tone [76,80].

### 3.6. Rheological Parameters of Goat Kids’ Meat

**Figure 4:**
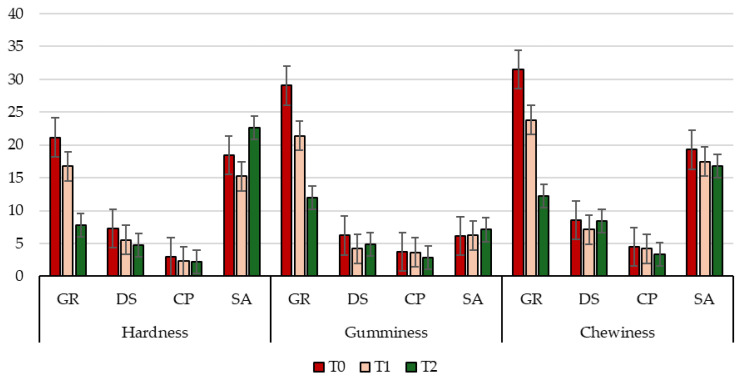
Changes in the main parameters of the meat texture of *Longissimus thoracis et lumborum* (LTL) goat muscles from the four groups of kids (GR, *Garganica*; DS, *Derivata di Siria*; CP, *Capra di Potenza*; SA, *Saanen*) during the aging period.

**Figure 5:**
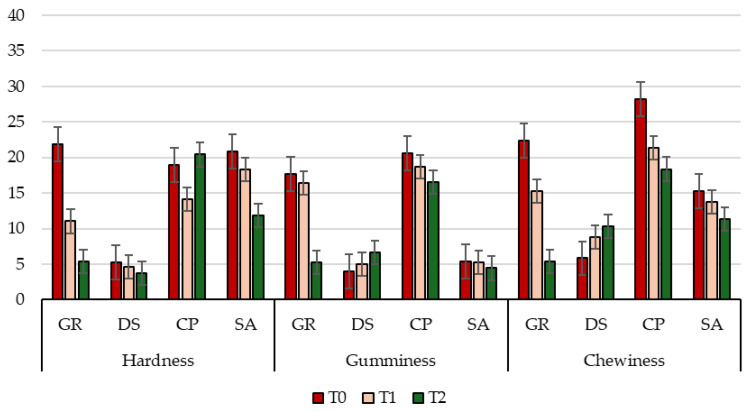
Changes in the main parameters of the meat texture of *Quadriceps femoris* (QF) goat muscles from the four groups of kids (GR, *Garganica*; DS, *Derivata di Siria*; CP, *Capra di Potenza*; SA, *Saanen*) during the aging period.

Rheological properties of meat, especially hardness, gumminess, chewiness, and elasticity, exert a decisive influence on the consumer’s sensory perception, impacting key quality attributes including tenderness, juiciness, and ease of mastication [81]. Overall, the results obtained from the TPA test analyses, performed on raw goat LTL and QF muscle cut samples belonging to the four different kids’ groups (GR, DS, CP, and SA), highlighted a positive effect of aging on all the tested parameters, particularly hardness, gumminess, and chewiness (Figure 4 and Figure 5). These findings suggest a sensory improvement in meat texture properties as the aging period progresses, which can be attributed to the series of physical and biochemical reactions occurring during the wet ageing process. Among these, the hydrolysis of myofibrillar proteins by endogenous enzymes, such as calpains and cathepsins, is known for releasing small peptides (e.g., carnosine) and free amino acids (FAAs), which contribute to enhanced tenderness and flavor [81]. Concerning the genetic breed effect, data revealed that meat from kids of local genetic types, despite some differences among them, exhibited more favorable rheological characteristics and higher consumer acceptability compared to the selected breed, which was characterized by greater tenderness, homogeneity across muscle cuts, and reduced chewiness. These features make the kids of the local breeds particularly suitable for high-quality traditional productions and for highlighting authentic regional flavors. Specifically, regarding hardness, a key parameter influencing perceived softness [82], a progressively significant (*p* < 0.01; Table S4) decrease was observed over time in GR and DS kids for both analyzed muscles. CP showed a significant (*p* < 0.01; Table S4) increase at the end of the aging period in the QF anatomical cut. Conversely, SA exhibited a significant (*p* < 0.01; Table S4) increase in hardness value in LTL at T2, potentially compromising the final perception of tenderness [83]. The observed increases could be attributed to the marked reduction in muscle tissue water content from T1 to T2 [18], as shown in Table 3. Concerning gumminess and chewiness, both related to the effort required for mastication [82], data showed clear breed-related differences, muscle-specific patterns, and a progressive decline over the aging period. Among groups of kids, GR consistently displayed the highest values for both parameters in both muscles and at all time points, indicating a firmer and more resistant texture. In contrast, DS and CP exhibited markedly lower values, reflecting a more tender structure and, consequently, a potentially more favorable sensory profile. Finally, SA showed intermediate levels, higher than DS and CP but lower than GR, confirming its overall tougher texture compared with the kids of local breeds. Muscle comparison revealed that the LTL muscle cut generally exhibited higher gumminess and chewiness than QF in all groups of kids, except CP, where QF values were significantly (*p* < 0.01; Table S4) higher than LTL, highlighting a distinctive resistance of the hind leg muscle in this genotype. Over the aging period (T0 to T2), all breeds and muscles experienced a progressive decrease in gumminess and chewiness, indicating effective tenderization during the wet aging [83]. The reduction was particularly pronounced in GR and in the QF of CP, suggesting a strong response to aging despite initially high toughness. Conversely, SA meat showed a less marked decline, especially in the LTL muscle, maintaining relatively high values throughout the aging period and confirming a lower susceptibility to tenderization, which may negatively affect consumer perception of meat palatability [82]. Also, adhesiveness (Table S4), which reflects the meat’s tendency to stick on the palate surface [84], showed significant differences among groups of kids. Local genetic types, particularly GR, exhibited more uniform and generally lower values, indicative of less sticky and more pleasant meat during mastication. Regarding springiness, a texture parameter highly related to intramuscular fat content [85], values remained relatively stable over time and across groups of kids. However, SA meat exhibited significantly lower values (*p* < 0.01; Table S4), particularly in the LTL muscle, accompanied by a concurrent increase in hardness over the same period (T2), which could potentially compromise the perceived firmness and juiciness during mastication [81]. Finally, concerning resilience (Table S4), which reflects the tissue’s ability to regain its original shape following compression [82], fluctuations were observed in all kids. An interesting exception was noted in CP, where the QF muscle showed a consistent increase over time, indicating that a more compact structure may enhance chewing comfort and cutting of meat [86].

The results obtained from the Warner–Bratzler shear force (WBSF) test performed on raw meat samples collected from the LTL and QF muscle cuts of the four different breeds (GR, DS, CP, and SA) are presented in Table 7. Overall, data clearly show that kid goat meat tenderness changes over time, depending on both breed and muscle type. Notably, in the back muscles (LTL), tenderness markedly increased over the seven-day aging period in CP, with a linear decrease in shear force observed over time. This reduction reflects the efficient degradation of myofibrillar structures and collagen by endogenous muscle proteinases, a process promoted during post-mortem aging [7]. Tenderness also increased in GR from T0 to T2, and to a lesser extent, in SA and DS over the same period, as evidenced by the decrease in shear force observed at the end of the aging period in all breeds (Table 7). In the thigh muscles (QF), on the other hand, tenderness decreased significantly (*p* < 0.05) and linearly over time in GR and CP, where a progressive increase in shear force was observed. This decline may be attributed to alterations in muscle structure, reduced enzymatic activity impacting the proteolysis of myofibrillar proteins [87], or a marked reduction in intramuscular water content (a_w_), as further supported by the a_w_ measurements presented in Table 2. Conversely, a linear but non-significant increase in tenderness was observed in DS and SA, suggesting a gradual softening of meat over time. This effect is desirable from a consumer perspective and indicates a positive response to wet aging [7]. In summary, the LTL muscle, despite exhibiting more variable patterns, appears to respond more effectively to the wet aging technique than the QF muscle, consistently showing improved tenderness at the end of the storage (T2) in all breeds. The present results underscore the importance of tailoring aging times and methods to the specific characteristics of each product and muscle type.

### 3.7. Aromatic Traits of Goat Kids’ Raw and Cooked Meat

**Figure 6:**
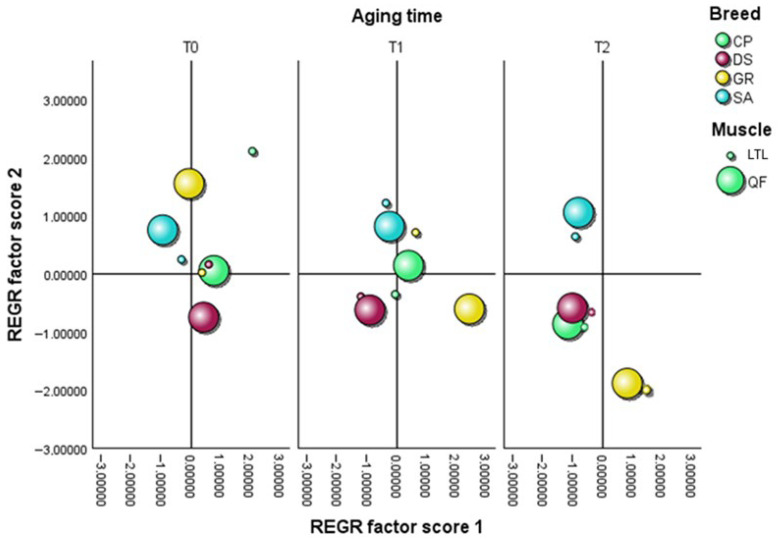
Principal component analysis (PCA) of E-nose odor data performed on raw samples from *Longissimus thoracis et lumborum* (LTL) and *Quadriceps femoris* (QF) muscle cuts belonging to *Garganica* (GR), *Derivata di Siria* (DS), *Capra di Potenza* (CP), and *Saanen* (SA) kids breeds.

**Figure 7:**
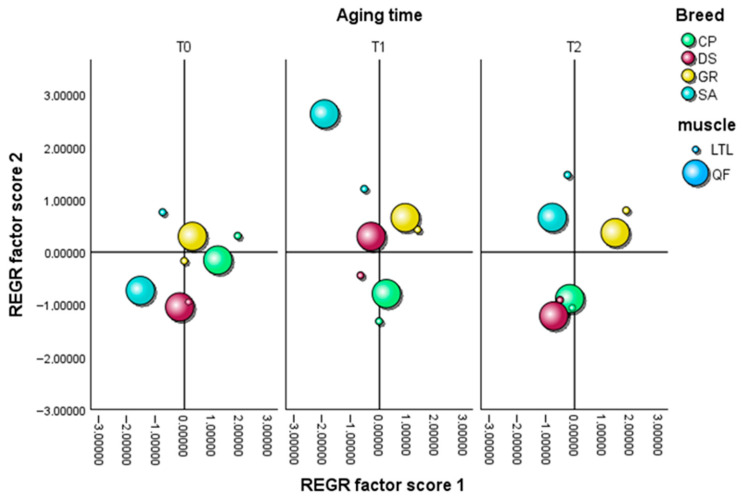
Principal component analysis (PCA) of E-nose odor data performed on cooked samples from *Longissimus thoracis et lumborum* (LTL) and *Quadriceps femoris* (QF) muscle cuts belonging to *Garganica* (GR), *Derivata di Siria* (DS), *Capra di Potenza* (CP), and *Saanen* (SA) kids breeds.

The multivariate analysis of the E-nose dataset, as represented by the PCA score plots (Figure 6 and Figure 7), explained a total variance of 76.950% in raw samples (PC1 = 59.636%, PC2 = 17.314%) and of 87.594% in cooked cuts (PC1 = 66.902%, PC2 = 20.692%), highlighting the volatile profile differences according to breed, muscle type, aging time, and thermal processing. Notably, in raw meat samples (Figure 6), all breeds showed overlapping distributions at T0, suggesting comparable aromatic profiles immediately after slaughter with no clear differences, except for the GR QF and CP LTL samples, which deviated from the central cluster. As aging progressed to T1, the SA, DS, and CP samples remained clustered around the origin, suggesting a more stable aromatic composition, while a greater dispersion became evident in the GR group, which shifted towards positive values of both factors 1 and 2. By T2, all samples shifted away from the center of the plot, reflecting an increased divergence in volatile profiles as aging progressed, with CP and DS closely clustered with each other, indicating comparable aromatic trajectories, whereas GR and SA diverged, showing a broader distribution along both axes. In cooked samples (Figure 7), the variance among groups was more pronounced than that observed in raw meat. At T0, all breeds were already clearly distinct, reflecting specific aromatic characteristics. A closer similarity was observed between the local breeds of GR and CP, which clustered nearer to the center of the plot and each other compared with DS and SA. At T1, a more pronounced distance among breeds emerged, reflecting distinct trajectories of aromatic evolution, with SA appearing the most distant from the origin, particularly in the QF muscle. By T2, all breeds shifted further away from the plot center, exhibiting broader dispersion. However, it is important to note that, despite their distance from the origin, the CP and DS samples remained closely clustered to each other, suggesting comparable patterns of volatile development that appeared more stable and less susceptible to oxidative changes at the end of the aging period. These results are also supported by the TBAR values shown in Table 4, as well as by the responses of the main E-nose sensors recorded during the aging period (Figures S1 and S2; Supplementary Materials).

## 4. Conclusions

The study demonstrates that wet aging for up to seven days can be effectively applied to improve tenderness and quality of meat, without negatively affecting nutritional value or oxidative stability, particularly in the *Longissimus thoracis* et *lumborum* muscle. The wet aging methods significantly improved meat tenderness and reduced chewiness across all breeds, particularly in the *Longissimus thoracis* et *lumborum* muscle. In particular, the most pronounced effect of this method was observed in the meat of local breeds, which yielded not only more tender, palatable, and flavorful meat with lower hardness, gumminess, and chewiness but also a more stable aromatic profile throughout the aging period, thereby reducing the likelihood of off-flavor development at the end. Overall, these findings highlight the potential of wet aging to improve sensory consistency and support the valorization of local breeds while also demonstrating the suitability of local goat breeds for premium and niche meat productions, particularly in the context of biodiversity preservation. Although the sample size represents a limitation of the present study, the results provide a basis for future research with larger populations aimed at further validating the observed breed- and aging-related effects. The results demonstrate the effectiveness of the wet aging methods as a post-mortem strategy to enhance their quality, providing a scientific foundation for the inclusion of these breeds in quality certification programs and territorial marketing initiatives. This approach may represent a valuable strategy to counteract genetic erosion while also enhancing the diversity and quality of goat meat available to consumers.

## Figures and Tables

**Table 1:** Goat kids’ carcasses parameters post-slaughter presented as biological mean ± standard error in *Garganica* (GR), *Derivata di Siria* (DS), *Capra di Potenza* (CP), and *Saanen* (SA) groups (n = 10 animals per group).

Breed	Dressing Percentage (%)	pH LTL 30 min	pH QF 30 min	pH LTL 24 h	pH QF 24 h	*p* LTL	*p* QF
GR	58.59 ± 2.10 ^X^	7.37 ± 0.28 ^Y,A^	7.04 ± 0.29 ^A^	5.53 ± 0.14 ^X,B^	5.75 ± 0.35 ^X,x,B^	NS	***
DS	45.74 ± 3.29 ^Y^	7.25 ± 0.33 ^A^	6.76 ± 0.19 ^A^	5.89 ± 0.13 ^y,B^	6.15 ± 0.32 ^y,B^	***	***
CP	61.14 ± 5.48 ^X^	6.85 ± 0.48 ^X,x,A^	6.83 ± 0.50 ^A^	6.14 ± 0.25 ^x,Y,B^	6.27 ± 0.37 ^Y,B^	***	***
SA	53.60 ± 1.26	7.34 ± 0.23 ^y,A^	6.69 ± 0.19 ^A^	6.10 ± 0.17 ^Y,B^	6.04 ± 0.24 ^B^	***	***

Values are expressed as biological mean ± standard errors. Different lowercase (x, y = *p* < 0.05) or uppercase letters (X, Y = *p* < 0.01) in the same column indicate significant differences among breeds. Different uppercase letters (A, B = *p* < 0.01) in the same row indicate significant differences among pH time measurements post-slaughter (30 min and 24 h) within the same muscle. (***) *p* < 0.001 indicates significant or not significant (NS) differences between LTL and QF muscles within the same breed.

**Table 2:** Biological mean values (±standard errors) of pH and a_w_ in the *Longissimus thoracis et lumborum* (LTL) and *Quadriceps femoris* (QF) muscle cuts from the four groups of kids (GR, *Garganica*; DS, *Derivata di Siria*; CP, *Capra di Potenza*; SA, *Saanen*) during the aging period (n = 10 animals per group).

				Aging Time			Effect	
Items	Breed	Cut	T0 (0 d)	T1 (3 d)	T2 (7 d)	T	B	T × B
a_w_	GR	LTL	0.982 ± 0.01	0.985 ± 0.00	0.984 ± 0.00			
		QF	0.984 ± 0.00	0.985 ± 0.00 ^a^	0.979 ± 0.02 ^b^			
	DS	LTL	0.982 ± 0.004	0.983 ± 0.001	0.982 ± 0.002			
		QF	0.983 ± 0.002	0.983 ± 0.001	0.983 ± 0.001			
	CP	LTL	0.982 ± 0.004	0.983 ± 0.001	0.982 ± 0.002			
		QF	0.982 ± 0.002	0.982 ± 0.001	0.980 ± 0.001			
	SA	LTL	0.982 ± 0.003	0.983 ± 0.001	0.982 ± 0.001			
		QF	0.982 ± 0.007	0.982 ± 0.003	0.980 ± 0.002	NS	NS	NS
pH	GR	LTL	5.73 ± 0.15	5.70 ± 0.09	5.72 ± 0.19			
		QF	5.71 ± 0.15	5.68 ± 0.08	5.75 ± 0.15			
	DS	LTL	5.76 ± 0.20	5.67 ± 0.17	5.76 ± 0.23			
		QF	5.75 ± 0.27	5.78 ± 0.14	5.78 ± 0.10			
	CP	LTL	5.91 ± 0.21	5.88 ± 0.12	6.00 ± 0.13			
		QF	5.68 ± 0.18	5.74 ± 0.13	6.00 ± 0.11			
	SA	LTL	5.59 ± 0.09	5.58 ± 0.08	5.92 ± 0.06			
		QF	5.52 ± 0.10	5.58 ± 0.16	5.87 ± 0.07	NS	*	NS

Values are expressed as biological mean ± standard errors. Different lowercase letters (a, b = *p* < 0.05)in the same row indicate significant differences among storage times within the same muscle. In the present table, no significant differences (NS) were detected between LTL and QF within the same breed during the wet aging period. On the right, NS: not significant; (*) *p* < 0.05 indicates the effects of aging time, breed, and the time × breed interaction. ANOVA was performed by a general linear model (GLM), including the fixed effect of aging time (T), the breeds (B), and their interaction (T × B).

**Table 3:** Biological mean values (±standard errors) of moisture, fat, protein, and salt content (%) in the *Longissimus thoracis et lumborum* (LTL) and *Quadriceps femoris* (QF) muscle cuts from the four groups of kids (GR, *Garganica*; DS, *Derivata di Siria*; CP, *Capra di Potenza*; SA, *Saanen*) during the aging period (n = 10 animals per group).

				Aging Time			Effect	
Items	Breed	Cut	T0 (0 d)	T1 (3 d)	T2 (7 d)	T	B	T × B
Moisture, %	GR	LTL	74.17 ± 1.74 ^a,y^	75.88 ± 0.87 ^b^	74.74 ± 0.40			
		QF	74.80 ± 1.25	76.24 ± 0.64	75.40 ± 0.67			
	DS	LTL	75.59 ± 1.08	75.74 ± 1.13	76.55 ± 0.46			
		QF	76.02 ± 0.69	76.39 ± 0.86	76.30 ± 0.86			
	CP	LTL	75.52 ± 0.85 ^x^	75.02 ± 1.34	75.51 ± 0.42			
		QF	75.50 ± 2.53	74.96 ± 0.72	76.30 ± 0.79			
	SA	LTL	75.94 ± 1.02 ^x^	74.78 ± 1.04	76.48 ± 1.15			
		QF	75.14 ± 0.29	75.45 ± 0.79	76.41 ± 0.78	**	*	NS
Fat, %	GR	LTL	3.16 ± 1.58 ^a,X^	2.21 ± 0.37 ^b^	2.22 ± 0.91 ^b^			
		QF	2.35 ± 1.19	1.99 ± 0.89	1.97 ± 0.54			
	DS	LTL	2.31 ± 0.58 ^Y^	2.6 ± 0.62	2.34 ± 0.55			
		QF	1.98 ± 0.90	1.81 ± 0.52	1.91 ± 0.95			
	CP	LTL	3.31 ± 1.04	2.53 ± 1.13	2.33 ± 1.23			
		QF	3.03 ± 1.16	2.40 ± 0.28	1.67 ± 0.19			
	SA	LTL	2.39 ± 1.90	1.72 ± 0.28	1.76 ± 0.42			
		QF	2.04 ± 0.84	2.05 ± 0.40	1.73 ± 0.72	**	NS	NS
Protein, %	GR	LTL	16.82 ± 1.62 ^X^	17.99 ± 0.78	17.01 ± 0.76			
		QF	17.73 ± 0.74 ^x^	17.81 ± 0.63	17.54 ± 0.37 ^x^			
	DS	LTL	17.06 ± 0.42 ^X^	18.01 ± 1.49 ^X^	17.43 ± 0.69 ^x^			
		QF	17.55 ± 0.78 ^x^	17.69 ± 1.24 ^X^	17.37 ± 1.38 ^X^			
	CP	LTL	17.56 ± 0.36 ^x^	17.51 ± 1.03 ^X^	18.56 ± 1.15			
		QF	17.43 ± 0.91 ^X^	17.56 ± 0.54 ^x^	17.85 ± 0.67 ^x^			
	SA	LTL	19.32 ± 0.33 ^y,Y^	19.71 ± 0.07 ^Y^	18.96 ± 0.12 ^y^			
		QF	19.11 ± 0.11 ^Y,y^	19.56 ± 0.04 ^y,Y^	19.75 ± 0.27 ^y,Y^	NS	***	NS
NaCl, %	GR	LTL	0.57 ± 0.04	0.66 ± 0.17	0.80 ± 0.24			
		QF	0.53 ± 0.04	0.66 ± 0.17	0.68 ± 0.19			
	DS	LTL	0.64 ± 0.10	0.82 ± 0.13	0.74 ± 0.11			
		QF	0.79 ± 0.12	0.80 ± 0.16	0.77 ± 0.13			
	CP	LTL	0.61 ± 0.08	0.75 ± 0.10	0.71 ± 0.10			
		QF	0.66 ± 0.07	0.79 ± 0.12	0.73 ± 0.13			
	SA	LTL	0.85 ± 0.05	0.73 ± 0.12	0.76 ± 0.16			
		QF	0.89 ± 0.10	0.77 ± 0.09	0.79 ± 0.07	NS	**	NS

Values are expressed as biological mean ± standard errors. Different lowercase letters (a, b = *p* < 0.05) in the same row indicate significant differences among storage times within the same muscle. Different lowercase (x, y = *p* < 0.05) or uppercase letters (X, Y = *p* < 0.01) in the same column indicate significant differences among breeds for the same muscle at a given storage time. In the present table, no significant differences (NS) were detected between LTL and QF within the same breed during the wet aging period. On the right, NS: not significant; (*) *p* < 0.05; (**) *p* < 0.01; and (***) *p* < 0.001 indicate the effects of aging time, breed, and the time × breed interaction. ANOVA was performed by a general linear model (GLM), including the fixed effect of aging time (T), the breeds (B), and their interaction (T × B).

**Table 4:** Nutritional index of intramuscular fat from kid goats as a function of aging time (T) and breed (B) in the *Longissimus thoracis et lumborum* muscle cut from the four groups of kids (GR, *Garganica*; DS, *Derivata di Siria*; CP, *Capra di Potenza*; SA, *Saanen*) wet-aged for 7 days (n = 10 animals per group).

		Aging Time			Effect		
Items	Breed	T0 (0 d)	T1 (3 d)	T2 (7 d)	T	B	T × B
P/S	GR	0.14 ± 0.05 ^X^	0.19 ± 0.02 ^Y^	0.19 ± 0.07 ^Y^			
	DS	0.18 ± 0.00 ^x,a^	0.19 ± 0.04 ^X,b^	0.48 ± 0.07 ^X,b^			
	CP	0.29 ± 0.07	0.21 ± 0.06	0.30 ± 0.12			
	SA	0.41 ± 0.10 ^Y,y^	0.34 ± 0.20	0.31 ± 0.03	NS	**	**
n6/n3	GR	4.01 ± 1.12 ^X^	3.91 ± 0.56 ^X^	3.72 ± 0.76 ^X^			
	DS	11.39 ± 0.49 ^Y^	10.78 ± 0.38 ^Y^	11.72 ± 1.31 ^Y^			
	CP	4.07 ± 1.07 ^X^	5.26 ± 0.34 ^X^	4.79 ± 0.16 ^X^			
	SA	16.59 ± 0.55 ^Z^	16.98 ± 0.76 ^Z^	16.48 ± 0.61 ^Z^	NS	***	NS
TI	GR	1.86 ± 0.17	1.83 ± 0.21	1.78 ± 0.05			
	DS	1.96 ± 0.01 ^x^	1.82 ± 0.03	1.45 ± 0.07			
	CP	1.45 ± 0.10 ^y^	1.58 ± 0.05	1.44 ± 0.03			
	SA	1.66 ± 0.07 ^y^	1.87 ± 0.13	1.88 ± 0.12	NS	**	NS
AI	GR	1.14 ± 0.19 ^x^	1.11 ± 0.10	1.08 ± 0.11			
	DS	1.07 ± 0.11	1.14 ± 0.19 ^a^	0.75 ± 0.07 ^b^			
	CP	0.68 ± 0.03 ^y^	0.93 ± 0.05	0.80 ± 0.07			
	SA	0.92 ± 0.07	1.11 ± 0.09	1.12 ± 0.12	NS	**	NS
h/H	GR	1.36 ± 0.22 ^X^	1.39 ± 0.12	1.42 ± 0.12			
	DS	1.34 ± 0.09 ^X,a^	1.31 ± 0.06 ^a^	1.84 ± 0.10 ^x,b^			
	CP	2.07 ± 0.11 ^Y,x,a^	1.55 ± 0.08 ^b^	1.77 ± 0.12			
	SA	1.54 ± 0.08 ^y^	1.34 ± 0.14	1.33 ± 0.11 ^y^	NS	***	**
SI	GR	1.08 ± 0.08 ^x^	1.07 ± 0.12 ^X^	1.08 ± 0.06			
	DS	0.87 ± 0.03 ^X^	1.01 ± 0.00	0.93 ± 0.02 ^x^			
	CP	1.21 ± 0.08 ^Y^	1.18 ± 0.00 ^X^	1.21 ± 0.06 ^X,y^			
	SA	0.82 ± 0.05 ^X,y^	0.78 ± 0.05 ^Y^	0.80 ± 0.04 ^Y^	NS	***	NS
NV	GR	0.81 ± 0.12 ^Y^	0.79 ± 0.07	0.78 ± 0.05			
	DS	0.82 ± 0.05 ^Y^	0.83 ± 0.03	0.66 ± 0.03			
	CP	0.54 ± 0.02 ^X,x^	0.70 ± 0.03	0.64 ± 0.02 ^x^			
	SA	0.77 ± 0.04 ^y^	0.87 ± 0.05	0.87 ± 0.07 ^y^	NS	***	NS

Values are expressed as biological mean ± standard errors. Different lowercase (a, b = *p* < 0.05) letters in the same row indicate significant differences among aging times (0, 3, and 7 days) within the same breed. Different lowercase (x, y = *p* < 0.05) or uppercase letters (X, Y, Z = *p* < 0.01) in the same column indicate significant differences among breeds for the same aging time. On the right, NS: not significant; (**) *p* < 0.01 and (***) *p* < 0.001 indicate the effects of aging time, breed, and the time × breed interaction. ANOVA was performed by a general linear model (GLM), including the fixed effect of aging time (T), the breeds (B), and their interaction (T × B).

**Table 5:** Nutritional index of intramuscular fat from kid goats as a function of aging time (T) and breed (B) in the *Quadriceps femoris* muscle cut from the four groups of kids (GR, *Garganica*; DS, *Derivata di Siria*; CP, *Capra di Potenza*; SA, *Saanen*) wet-aged for 7 days (n = 10 animals per group).

		Aging Time			Effect		
Items	Breed	T0 (0 d)	T1 (3 d)	T2 (7 d)	T	B	T × B
P/S	GR	0.18 ± 0.03 ^X^	0.35 ± 0.23	0.32 ± 0.10 ^X^			
	DS	0.24 ± 0.07 ^X^	0.28 ± 0.02	0.20 ± 0.11 ^X^			
	CP	0.14 ± 0.02 ^X^	0.25 ± 0.01	0.30 ± 0.12 ^X^			
	SA	0.54 ± 0.10 ^Y,a^	0.24 ± 0.06 ^b^	0.62 ± 0.03 ^Y,a^	NS	***	***
n-6/n-3	GR	3.44 ± 0.14 ^X^	3.37 ± 0.04 ^X,x^	3.86 ± 0.73 ^X^			
	DS	11.30 ± 1.20 ^Y,a^	12.53 ± 0.09 ^Y,b^	11.65 ± 0.43 ^Y,a^			
	CP	4.63 ± 0.29 ^X^	4.84 ± 0.12 ^X,y^	4.79 ± 0.16 ^X^			
	SA	15.43 ± 1.20 ^Z,a^	15.72 ± 0.41 ^Z,a^	17.09 ± 0.39 ^Z,b^	NS	***	*
TI	GR	1.75 ± 0.41	1.57 ± 0.23 ^x^	1.53 ± 0.32			
	DS	1.77 ± 0.26	1.81 ± 0.08	1.66 ± 0.06			
	CP	1.77 ± 0.01	1.57 ± 0.05 ^x^	1.44 ± 0.06	**	NS	*
	SA	1.43 ± 0.03 ^A^	2.05 ± 0.02 ^B,y^	1.36 ± 0.07 ^A^			
AI	GR	1.07 ± 0.29	0.86 ± 0.24	0.87 ± 0.33			
	DS	1.07 ± 0.13	1.11 ± 0.11	1.02 ± 0.05			
	CP	1.08 ± 0.02	0.90 ± 0.00	0.80 ± 0.12			
	SA	0.81 ± 0.02 ^A^	1.34 ± 0.02 ^B^	0.72 ± 0.11 ^A^	*	NS	**
h/H	GR	1.41 ± 0.26	1.80 ± 0.54 ^x^	1.81 ± 0.52			
	DS	1.36 ± 0.18	1.31 ± 0.07	1.37 ± 0.06			
	CP	1.40 ± 0.02	1.61 ± 0.03	1.77 ± 0.21			
	SA	1.61 ± 0.06	1.11 ± 0.02 ^y,a^	1.94 ± 0.22 ^b^	NS	*	*
SI	GR	1.09 ± 0.19	1.06 ± 0.08	1.17 ± 0.18 ^X^			
	DS	0.99 ± 0.08	0.88 ± 0.01 ^x^	1.08 ± 0.01			
	CP	1.14 ± 0.03 ^x^	1.13 ± 0.06 ^X,y^	1.21 ± 0.10 ^X^			
	SA	0.90 ± 0.07 ^y^	0.80 ± 0.08 ^Y^	0.86 ± 0.03 ^Y^	*	***	NS
NV	GR	0.79 ± 0.17	0.67 ± 0.13 ^X^	0.66 ± 0.20			
	DS	0.82 ± 0.09	0.87 ± 0.03	0.79 ± 0.04			
	CP	0.75 ± 0.01	0.69 ± 0.01 ^X^	0.64 ± 0.04			
	SA	0.69 ± 0.01 ^A^	1.00 ± 0.02 ^Y,B^	0.63 ± 0.07 ^A^	**	*	**

Values are expressed as biological mean ± standard errors. Different lowercase (a, b = *p* < 0.05) or uppercase letters (A,B = *p* < 0.01) in the same row indicate significant differences among aging times (0, 3, and 7 days) within the same breed. Different lowercase (x, y = *p* < 0.05) or uppercase letters (X, Y, Z = *p* < 0.01) in the same column indicate significant differences among breeds for the same aging time. On the right, NS: not significant; (*) *p* < 0.05; (**) *p* < 0.01; and (***) *p* < 0.001 indicate the effects of aging time, breed, and the time × breed interaction. ANOVA was performed by a general linear model (GLM), including the fixed effect of aging time (T), the breeds (B), and their interaction (T × B).

**Table 6:** Biological mean values ± standard errors of TBAR_s_ in the *Longissimus thoracis et lumborum* (LTL) and *Quadriceps femoris* (QF) muscle cuts from the four groups of kids (GR, *Garganica*; DS, *Derivata di Siria*; CP, *Capra di Potenza*; SA, *Saanen*) during the aging period (n = 10 animals per group).

			Aging Time			Effect	
Breed	Cut	T0 (0 d)	T1 (3 d)	T2 (7 d)	T	B	T × B
GR	LTL	0.131 ± 0.051	0.133 ± 0.060	0.190 ± 0.045 ^x^			
	QF	0.124 ± 0.092	0.130 ± 0.033	0.171 ± 0.025			
DS	LTL	0.037 ± 0.006	0.065 ± 0.014	0.078 ± 0.027 ^X^			
	QF	0.030 ± 0.024 ^x^	0.039 ± 0.010 ^X^	0.040 ± 0.020 ^X^			
CP	LTL	0.060 ± 0.022	0.090 ± 0.028	0.110 ± 0.040 ^X^			
	QF	0.070 ± 0.035	0.090 ± 0.031	0.100 ± 0.034			
SA	LTL	0.203 ± 0.040 ^A^	0.264 ± 0.118	0.426 ± 0.400 ^B,Y,y^			
	QF	0.235 ± 0.066 ^y^	0.281 ± 0.167 ^Y^	0.299 ± 0.293 ^Y^	NS	***	NS

Values are expressed as biological mean ± standard errors. Different uppercase letters (A, B = *p* < 0.01) in the same row indicate significant differences among storage times within the same muscle. Different lowercase (x, y = *p* < 0.05) or uppercase letters (X, Y = *p* < 0.01) in the same column indicate significant differences among breeds for the same muscle at a given storage time. In the present table, no significant differences (NS) were detected between LTL and QF within the same breed during the wet aging period. On the right, NS: not significant; (***) *p* < 0.001 indicates the effects of aging time, breed, and the time × breed interaction. ANOVA was performed by a general linear model (GLM), including the fixed effect of aging time (T), the breeds (B), and their interaction (T × B).

**Table 7:** Biological mean values (±standard errors) of Warner–Bratzler shear force (WBSF) measured on *Longissimus thoracis et lumborum* (LTL) and *Quadriceps femoris* (QF) muscle cuts from the four groups of kids (GR, *Garganica*; DS, *Derivata di Siria*; CP, *Capra di Potenza*; SA, *Saanen*) during the aging period (n = 10 animals per group).

			Aging Time			Effect	
Breed	Cut	T0 (0 d)	T1 (3 d)	T2 (7 d)	T	B	T × B
GR	LTL	131.21 ± 23.82	154.08 ± 24.14	96.50 ± 9.50			
	QF	94.49 ± 7.45 ^a^	190.50 ± 4.16	193.16 ± 0.72 ^b,x^			
		NS	NS	*			
DS	LTL	106.21 ± 13.58	143.41 ± 14.33	102.41 ± 9.28			
	QF	185.80 ± 3.16	152.19 ± 29.05	103.34 ± 22.35			
		NS	*	NS			
CP	LTL	190.49 ± 11.11	114.60 ± 8.28	87.15 ± 0.67			
	QF	100.17 ± 1.71 ^a^	139.72 ± 19.36	220.11 ± 3.24 ^x,b^			
		NS	NS	*			
SA	LTL	126.14 ± 2.95	133.91 ± 2.81	105.44 ± 1.44			
	QF	117.53 ± 8.34	83.43 ± 13.10	68.52 ± 9.41 ^y^			
		NS	NS	NS	NS	NS	NS

Values are presented as biological mean ± standard errors. Different lowercase letters (a, b = *p* < 0.05)in the same row indicate significant differences among storage times within the same muscle. Different lowercase letters (x, y = *p* < 0.05) in the same column indicate significant differences among breeds for the same muscle at a given storage time. Asterisks ((*) *p* < 0.05) indicate significant differences between LTL and QF within the same breed at a given storage time, while NS denotes no significant difference. On the right, NS: not significant indicate the effect of aging time, breed, and the time × breed interaction. ANOVA was performed by a general linear model (GLM), including the fixed effect of aging time (T), the breeds (B), and their interaction (T × B).

## Data Availability

Data are available within the article. The authors confirm that the data supporting the findings of this study are available within the article.

## Supplementary Materials

The following supporting information can be downloaded at https://www.mdpi.com/article/10.3390/ani16010115/s1, Table S1: Changes in the fatty acid composition (mg FA/100 g of meat) of intramuscular fat from kid goats in the *Longissimus thoracis et lumborum* muscle cut from the four kids breeds groups wet-aged for 7 days (GR, *Garganica*; DS, *Derivata di Siria*; CP, *Capra di Potenza*; SA, *Saanen*) as a function of the aging time (T) and breed (B) (n = 10 animals per group); Table S2: Changes in the fatty acid composition (mg FA/100 g of meat) of intramuscular fat from kid goats in the *Quadriceps femoris* muscle cut from the four goat breeds groups wet-aged for 7 days (GR, *Garganica*; DS, *Derivata di Siria*; CP, *Capra di Potenza*; SA, *Saanen*) as a function of the aging time (T) and breed (B) (n = 10 animals per group); Table S3: Biological mean values (±standard errors) of color parameters (L*, a*, b*, chroma, and hue) in the *Longissimus thoracis et lumborum* (LTL) and *Quadriceps femoris* (QF) muscle cuts from the four goat breeds groups (GR, *Garganica*; DS, *Derivata di Siria*; CP, *Capra di Potenza*; SA, *Saanen*) during the aging period (n = 10 animals per group); Table S4: Biological mean values (±standard errors) of texture parameters in the *Longissimus thoracis et lumborum* (LTL) and *Quadriceps femoris* (QF) muscle cuts from the four goat breeds (GR, *Garganica*; DS, *Derivata di Siria*; CP, *Capra di Potenza*; SA, *Saanen*) during the aging period (n = 10 animals per group); Figure S1: Changes of the main E-nose sensors in raw goat meat from *Longissimus thoracis et lumborum* (LTL) and *Quadriceps femoris* (QF) muscle cuts belonging to *Garganica* (GR), *Derivata di Siria* (DS), *Capra di Potenza* (CP), and *Saanen* (SA) breeds groups during the aging period (T0, 0 days; T1, 3 days; and T2, 7 days) (n = 10 animals per group); Figure S2: Changes of the main E-nose sensors in cooked goat meat from *Longissimus thoracis et lumborum* (LTL) and *Quadriceps femoris* (QF) muscle cuts belonging to *Garganica* (GR), *Derivata di Siria* (DS), *Capra di Potenza* (CP), and *Saanen* (SA) breeds groups during the aging period (T0, 0 days; T1, 3 days; and T2, 7 days) (n = 10 animals per group).

## Abbreviations

The following abbreviations are used in this manuscript:

AI	Atherogenic Index
TI	Thrombogenic Index
SI	Meat Softness Index
NV	Nutritional Value of lipids
h/H	Ratio of hypo- and hypercholesterolemic fatty acids
MUFAs	Monounsaturated Fatty Acids
PUFAs	Polyunsaturated Fatty Acids
SFAs	Saturated Fatty Acids
CLA	Conjugated Linoleic Acid
EPA	Eicosapentaenoic Acid
DHA	Docosahexaenoic Acid
TBARs	Thiobarbituric Acid Reactive Substances
IMF	Intramuscular Fat
GR	*Garganica*
DS	*Derivata di Siria*
CP	*Capra di Potenza*
SA	*Saanen*
QF	*Quadriceps Femoris*
LTL	*Longissimus Thoracis et Lumborum*
NS	Not Significant
PCA	Principal Component Analysis
TPA	Texture Profile Analysis
WBSF	Warner–Bratzler Shear Force test
a_w_	Water activity
