# Supplementary material for: Effect of Wet Aging on the Meat Quality of Two Cuts (Longissimus thoracis et lumborum and Quadriceps femoris) from Italian Local Goat Breeds Compared to the Saanen Cosmopolitan Breed
Source: Animals (Basel). 2025 Dec 31;16(1):115. doi: 10.3390/ani16010115 (PMC12785098; doi:10.3390/ani16010115)
Supplement: Supplementary file 1 [file animals-16-00115-s001.zip › animals-4021675-supplementary.pdf]

Supplementary materials

Tables

Table S1. Changes in the fatty acid composition (mg FA/100g of meat) of intramuscular fat from kid goats in the *Longissimus thoracis et lumborum* muscle cut from the four kids breeds groups wet-aged for 7 days (GR, *Garganica*; DS, *Derivata di Siria*; CP, *Capra di Potenza*; SA, *Saanen*) as a function of the aging time (T) and breed (B) (n = 10 animals per group).

		Aging Time			Effect		
**Items**	**Breed**	**T0 (0d)**	**T1 (3d)**	**T2 (7d)**	**T**	**B**	**T x B**
C10:0	GR	9.63±2.60	7.31±0.84	9.90±1.17^{X}			
	DS	10.65±2.73^{X}	12.66±1.64^{X,a}	7.39±1.80^{b}			
	CP	4.92±0.02^{Y,x}	6.81±0.86^{Y}	4.93±0.36^{Y}			
	SA	7.49±0.94^{y}	7.00±1.23	7.55±2.09^{X}	NS	***	*
C12:0	GR	29.15±14.24	21.31±5.11	22.23±8.56			
	DS	19.72±8.67	23.86±7.50	13.15±4.82			
	CP	10.72±1.89	17.56±2.53	13.40±1.98			
	SA	14.93±3.51	15.10±3.38	15.70±5.83	NS	*	NS
C14:0	GR	230.60±61.25^{x}	168.14±11.40	164.98±37.13			
	DS	153.80±42.87	189.88±31.12	110.75±26.49			
	CP	145.94±6.42^{y}	161.03±13.36	126.82±26.68			
	SA	137.75±20.47	124.51±12.61	126.54±25.17	NS	*	NS
C14:1	GR	9.37±3.19^{X}	7.36±2.67	7.86±3.31			
	DS	31.49±24.51^{Y}	7.16±1.47	4.04±0.48			
	CP	24.30±15.56	8.22±1.29	6.94±2.42			
	SA	5.07±0.24^{X}	3.85±0.50	3.85±0.02	*	NS	*
C15:0	GR	24.51±8.15^{x}	14.99±1.24	15.81±3.22			
	DS	8.89±5.94^{y}	13.88±2.70	9.43±1.94^{x}			
	CP	16.77±1.18	17.42±3.66	14.48±4.63			
	SA	16.78±1.72	13.87±1.34	14.00±3.14^{y}	NS	***	NS
C16:0	GR	823.05±133.96^{X}	548.29±51.96	541.94±48.62			
	DS	556.62±36.75	684.07±9.58	531.11±3.32			
	CP	678.29±10.76^{AY}	616.54±35.75^{B}	535.85±20.50			
	SA	577.15±11.22	438.91±37.57	450.54±20.54	NS	*	NS
C16:1	GR	72.37±15.90	47.85±11.77	48.91±13.15			
	DS	41.72±4.53	53.86±4.17	35.23±0.25			
	CP	69.63±6.93	63.45±9.34	52.53±11.31			
	SA	45.24±1.70	31.81±6.63	31.69±2.60	NS	*	NS
C17:0	GR	35.23±2.28	21.39±3.25	22.70±1.47			
	DS	20.64±1.84	23.95±2.42	18.03±2.47^{X}			
	CP	36.84±3.51	28.94±3.92	25.86±4.10^{Y}			
	SA	25.54±1.37	19.07±3.70	19.04±0.07^{Y}	NS	***	NS
C17:1	GR	31.53±9.84^{x,A}	14.41±3.20^{B}	14.28±2.27^{B}			
	DS	13.54±0.85^{y}	16.52±1.29	12.06±0.20^{X}			
	CP	24.49±2.42	23.34±5.00	22.41±5.78^{Y}			
	SA	17.62±1.57	12.00±2.92	11.28±0.19	NS	**	NS
C18:0	GR	384.13±127.75	273.30±51.90	275.18±78.36			
	DS	372.90±96.00	298.65±6.96	282.87±16.37			
	CP	487.46±82.92	279.57±13.13	258.11±22.31			
	SA	297.53±6.62	222.01±6.05	230.74±2.72	NS	NS	NS

C18:1*trans*	GR	33.63±21.84	26.10±17.49	31.84±12.71			
	DS	44.24±19.08	39.10±27.33	43.33±2.73			
	CP	20.24±5.08	33.57±13.36	31.97±2.59			
	SA	43.87±5.81	34.36±7.74	37.21±5.81	NS	**	NS
C18:1*cis*-n9	GR	1221.95±74.30^{x}	818.31±60.23^{X}	828.43±45.81^{Y}			
	DS	765.38±5.14^{Y}	939.45±14.89^{X,a}	723.06±8.87^{X,b}			
	CP	1333.35±42.65^{Xy}	994.58±17.85^{X}	904.68±64.74^{Y}			
	SA	674.57±65.40^{Y}	488.20±77.72^{Y}	513.95±31.62^{X}	NS	***	NS
C18:2*trans*-n6	GR	15.67±0.42^{X}	9.55±0.96^{x}	9.66±1.90^{x}			
	DS	9.59±3.00^{X,a}	7.10±0.99^{y,b}	5.98±0.34^{y,b}			
	CP	14.42±2.23^{X}	9.68±0.57^{x}	9.09±0.72^{x}			
	SA	5.58±0.49^{Y}	4.00±0.90^{y}	4.22±0.47^{y}	*	***	NS
C18:2*cis*-n6 (LA)	GR	121.88±19.93^{X}	116.24±9.35^{X}	107.39±30.27^{Xx}			
	DS	124.28±10.49^{X,A}	139.82±17.15^{X,A}	276.95±13.02^{Yx,B}			
	CP	211.66±24.68^{x}	139.49±25.84^{X}	157.78±51.78^{y}			
	SA	280.70±60.40^{Yy}	181.47±86.69^{Y}	174.15±4.40^{y}	NS	***	**
C18:3-n3 (ALA)	GR	27.45±6.56^{x}	22.34±2.06^{x}	22.12±7.30^{X}			
	DS	8.36±1.16^{Xy}	9.62±0.00^{y}	15.68±0.00			
	CP	37.90±12.48^{Y}	21.37±4.41^{x}	24.47±7.55^{X}			
	SA	12.27±1.83^{X}	8.33±3.14^{y}	8.22±0.09^{Y}	NS	***	NS
C18:2c9t11 (CLA)	GR	17.32±7.82^{x}	13.04±3.04^{x}	13.81±1.44			
	DS	20.39±5.02^{y}	25.13±6.41^{Yy}	18.16±3.65			
	CP	25.50±1.61	18.20±0.72	17.21±1.85			
	SA	12.84±0.89^{x}	9.27±1.24^{X}	9.35±0.62	NS	***	NS
C20:4 n6 (AA)	GR	24.89±17.91^{Y}	35.22±3.31^{x}	35.94±17.64^{Y}			
	DS	57.36±4.39^{Ay}	64.25±19.40^{A}	56.46±0.52^{B,X}			
	CP	81.74±12.83^{x}	50.49±17.75	71.31±40.30^{Y}			
	SA	126.91±34.95^{Xy}	76.50±48.44^{y}	71.51±6.21	*	***	*
C20:5 n3 (EPA)	GR	9.90±7.46^{y}	13.27±0.82	14.67±8.75			
	DS	4.03±1.00^{X}	4.90±2.38	11.78±3.36			
	CP	29.13±13.70^{Yx}	10.60±3.77	16.72±9.22			
	SA	7.65±2.69^{y}	4.19±2.98	4.00±0.50	NS	**	NS
C22:6 n3 (DHA)	GR	6.65±3.55	6.57±1.14	7.85±4.72			
	DS	4.13±0.30^{x}	4.98±1.31	9.22±2.81			
	CP	15.66±5.56^{y}	6.97±2.69	9.87±4.28			
	SA	4.92±1.94	2.97±1.87	2.86±0.34	NS	**	NS
∑SFA$_{s}$	GR	1574.87±130.72^{y}	1083.14±99.48	1079.93±27.96			
	DS	1190.64±4.85^{Y,A}	1297.37±24.21^{a}	1024.74±50.60^{Bb,x}			
	CP	1426.05±101.15^{x}	1157.29±38.32	1009.19±35.44^{x}			
	SA	1104.25±33.58	858.44±57.76	883.65±51.45^{y}	NS	***	NS
∑MUFA$_{s}$	GR	1372.96±84.81^{X}	917.48±92.62^{X}	936.26±56.06^{y}			
	DS	902.85±6.96	1061.78±18.76^{x}	827.83±7.22^{Yx}			
	CP	1480.20±25.65^{X}	1127.72±18.27^{X}	1023.76±79.92^{X}			
	SA	793.37±72.88^{Y}	575.01±93.75^{Yy}	602.71±39.81^{Y}	NS	***	NS
∑PUFA$_{s}$	GR	212.16±55.09^{X}	209.40±9.66	203.83±69.36^{X}			
	DS	216.50±2.14^{x,A}	240.81±42.95^{A}	487.45±43.41^{YB}			
	CP	403.79±75.47	245.04±56.58	297.05±115.38			
	SA	452.38±104.64^{Yy}	286.52±146.69	273.61±11.65	**	***	NS
Total PUFA$_{s}$ n-3	GR	44.66±17.83	42.79±3.24	45.25±20.71			
	DS	17.41±0.52^{Xx}	20.45±4.30	38.66±7.30			

Items	Breed	T0 (0d)	T1 (3d)	T2 (7d)	T	B	T x B
	CP	83.61±31.08^{Y}	39.43±11.04	51.55±21.19^{X}			
	SA	25.69±6.47^{y}	15.94±8.17	15.58±0.12^{Y}	NS	***	**
Total PUFA$_s$ n-6	GR	167.10±37.82^{X}	166.17±11.81^{x}	158.03±48.42^{x}			
	DS	198.11±2.66^{X,A}	219.31±38.77^{A,x}	446.80±35.85^{B,y}			
	CP	318.34±43.25^{x}	205.16±45.41^{x}	244.86±93.88^{x}			
	SA	424.74±97.88^{Yy}	269.31±138.05^{y}	256.75±11.48^{y}	NS	***	**

Values were expressed as mean ± standard errors. Abbreviations: ∑SFA$_s$, sum of saturated fatty acids; ∑MUFA$_s$, sum of monounsaturated fatty acids; ∑PUFA$_s$, polyunsaturated fatty acids. LA: linoleic acid; ALA: α-linolenic acid; CLA: conjugated linoleic acid; AA: arachidonic acid; EPA: eicosapentaenoic fatty acids, DHA: docosahexaenoic fatty acids. Different lowercase (a,b = $p < 0.05$) or uppercase letters (A,B = $p < 0.01$) in the same row indicate significant differences among aging times (0, 3, and 7 days) within the same breed. Different lowercase (x,y = $p < 0.05$) or uppercase letters (X,Y = $p < 0.01$) in the same column indicate significant differences among breeds for the same aging time. On the right NS: not significant; (*) p < 0.05; (**) p < 0.01; (***) p < 0.001 indicate the effects of aging time, breed, and the time × breed interaction.

Table S2. Changes in the fatty acid composition (mg FA/100g of meat) of intramuscular fat from kid goats in the *Quadriceps femoris* muscle cut from the four goat breeds groups wet-aged for 7 days (GR, *Garganica*; DS, *Derivata di Siria*; CP, *Capra di Potenza*; SA, *Saanen*) as a function of the aging time (T) and breed (B) (n = 10 animals per group).

		Aging Time			Effect		
**Items**	**Breed**	**T0 (0d)**	**T1 (3d)**	**T2 (7d)**	**T**	**B**	**T x B**
C10:0	GR	6.79±2.61	5.31±1.80	6.71±5.39			
	DS	7.22±1.38	8.00±2.32	5.23±0.23			
	CP	11.39±0.36	7.55±0.49	3.53±0.26			
	SA	6.73±0.50	10.14±2.18^{A}	4.22±1.35^{B}	*	NS	NS
C12:0	GR	20.15±4.91	13.55±3.76	15.67±10.85			
	DS	14.52±2.45	16.45±5.61	12.87±0.44			
	CP	28.51±1.64	18.78±2.39	9.60±1.42			
	SA	11.53±2.21^{a}	23.51±5.35^{Ab}	8.84±3.10^{Ba}	*	NS	NS
C14:0	GR	171.89±32.07	119.42±32.18 ^{x}	119.34±45.31			
	DS	134.28±1.49	129.09±21.29	129.26±1.98			
	CP	225.28±3.73	149.03±2.51^{x}	90.90±19.12			
	SA	99.05±13.12^{A}	178.31±3.46^{B,y}	81.63±16.72^{A}	*	NS	**
C14:1	GR	7.12±1.13	5.27±1.58	6.04±1.77			
	DS	5.10±0.92	4.00±0.36	5.35±0.73			
	CP	6.18±1.16	6.18±0.40	4.98±1.74			
	SA	5.38±0.31	6.18±1.16	4.27±0.73	NS	NS	NS
C15:0	GR	17.08±2.18	11.70±3.11^{X}	11.54±3.11			
	DS	11.11±1.45	9.60±1.87^{X}	10.67±2.17			
	CP	22.77±2.47	15.25±1.48^{x}	10.38±3.31			
	SA	11.70±1.70^{a}	19.44±1.22^{Yy,Ab}	10.44±2.33^{B}	NS	*	*
C16:0	GR	573.64±66.90	437.10±66.44^{X}	438.11±57.19			
	DS	524.37±65.05	473.83±11.50	497.71±30.88			
	CP	748.89±18.48	556.34±5.06	384.07±14.70			
	SA	411.77±34.55^{A}	565.98±11.71^{Y,B}	383.33±20.88^{A}	NS	***	NS
C16:1	GR	49.19±4.54	37.23±8.22	41.16±6.08			
	DS	40.79±1.66	31.42±0.76	44.67±3.06			
	CP	66.66±4.67	60.41±16.18	37.65±8.11			
	SA	38.83±2.22	45.32±8.83	31.93±3.38	NS	NS	NS
C17:0	GR	24.43±2.60	18.24±4.21	17.19±2.75			
	DS	19.26±0.78	15.93±1.76^{x}	19.19±2.61			
	CP	36.78±2.79^{x}	25.94±1.99	18.54±2.94			

	SA	16.39±1.50^{y,a}	23.83±1.81^{y,b}	15.27±1.10^{a}	NS	***	*
C17:1	GR	54.83±67.84	13.01±1.27	11.51±1.74			
	DS	15.07±3.39	10.53±0.29	15.48±0.96			
	CP	26.16±2.71	19.76±0.23	16.07±4.14			
	SA	13.67±1.63	17.51±1.46	13.78±0.04	NS	NS	NS
C18:0	GR	281.30±28.43	257.14±17.24	235.03±12.45			
	DS	215.35±14.33	202.77±10.57	197.94±11.76			
	CP	373.55±12.65	292.00±23.17	184.99±15.99			
	SA	195.61±28.29	236.57±7.50	201.47±18.62	NS	**	NS
C18:1*trans*	GR	31.36±31.94	32.95±20.52	22.35±11.55^{x}			
	DS	31.62±13.90	41.74±8.91	52.64±19.98^{y}			
	CP	24.65±13.41	41.46±5.92	22.91±1.85			
	SA	31.95±2.41	42.06±6.16	25.58±0.98	NS	*	NS
C18:1*cis*-n9	GR	872.89±63.01^{X}	700.79±69.85^{Xx}	738.78±71.64^{X}			
	DS	686.87±18.07^{X}	566.46±9.07^{x,a}	703.48±7.84^{X,b}			
	CP	1208.77±37.38^{X}	899.96±11.60^{Yy}	648.42±46.40^{X}			
	SA	502.62±27.32^{Y}	597.64±56.13^{Xy}	469.24±20.41^{Y}	NS	***	NS
C18:2*trans*-n6	GR	11.05±2.96^{X}	7.51±1.62	8.69±1.39^{X}			
	DS	3.09±2.81^{Y,a}	4.90±0.03	6.29±0.43^{b}			
	CP	11.29±0.77^{X}	8.26±0.96	6.51±0.52			
	SA	4.38±0.31^{Y}	5.37±0.96	4.07±0.02^{Y}	NS	***	NS
C18:2*cis*-n6 (LA)	GR	108.56±7.35^{X}	155.13±82.89	141.91±37.49 ^{X}			
	DS	134.12±30.17^{X}	148.23±1.10	108.06±5.80^{X}			
	CP	120.31±17.44^{X}	149.23±10.63	113.08±37.12^{X}			
	SA	249.88±63.12^{Y,A}	169.06±41.10^{B}	279.04±3.66^{Y,A}	NS	***	***
C18:3-n3 (ALA)	GR	24.72±0.24^{Xx}	29.14±10.78^{Xx}	24.87±4.75^{Y}			
	DS	8.71±1.98^{Y}	9.13±0.44^{Y}	7.13±0.89^{X}			
	CP	21.83±0.17	22.57±0.66^{y}	17.54±5.41^{Y}			
	SA	10.53±2.28^{y}	8.54±1.61^{Y}	11.50±0.35^{X}	NS	***	NS
C18:2c9t11 (CLA)	GR	16.25±2.82	11.95±2.53	12.37±3.30^{X}			
	DS	17.97±3.81^{x}	15.42±4.58	19.61±4.23^{Y}			
	CP	22.33±1.00	16.02±1.28	12.33±1.33			
	SA	9.51±1.02^{y}	11.83±1.15	8.69±0.03^{X}	NS	***	NS
C20:4 n6 (AA)	GR	31.18±3.84	59.90±41.53	55.33±18.84^{X}			
	DS	59.92±17.18	64.72±7.77	47.25±2.01^{X}			
	CP	35.80±7.26	60.80±3.23	51.11±28.88^{X}			
	SA	47.05±45.02^{A}	60.22±20.02^{A}	128.76±1.14^{YB}	**	**	**
C20:5 n3 (EPA)	GR	13.02±1.15^{a}	25.39±18.50^{Xx,b}	20.67±6.53^{x}			
	DS	4.52±0.64	5.05±1.03	3.64±0.77^{y}			
	CP	8.32±1.68	14.24±1.28^{y}	11.98±6.60			
	SA	8.19±2.90	3.74±1.34^{Y}	7.98±0.50	NS	***	NS
C22:6 n3 (DHA)	GR	6.66±1.15^{a}	13.01±9.27^{Xx,b}	9.82±3.17			
	DS	4.50±0.15	3.71±0.54^{y}	3.20±0.30			
	CP	6.26±1.50	9.01±1.19	7.07±3.06			
	SA	4.88±2.24	2.68±1.32^{Y}	4.58±0.39	NS	**	NS
∑SFA$_{s}$	GR	1127.55±128.60	894.19±84.76^{x}	874.94±108.76			
	DS	971.27±61.22	902.94±17.16	904.02±12.74			
	CP	1481.59±3.11	1094.20±17.65^{x}	723.33±25.40			
	SA	775.82±82.24^{A}	1078.64±5.44^{B,y}	728.07±23.30^{A}	**	**	NS

∑MUFAₛ	GR	1021.08 ± 120.47^{X}	796.17 ± 91.42^{Y}	824.92 ± 63.06^{X}			
	DS	784.24 ± 3.19	$658.61 \pm 1.37^{x,a}$	$825.69 \pm 8.94^{X,b}$			
	CP	1339.23 ± 25.00^{X}	1034.06 ± 33.43^{Xy}	733.76 ± 57.28^{X}			
	SA	596.17 ± 32.29^{Y}	713.77 ± 72.18^{Y}	552.35 ± 18.57^{Y}	NS	***	NS
∑PUFAₛ	GR	201.38 ± 12.18^{X}	299.63 ± 167.02	270.13 ± 67.43^{X}			
	DS	225.29 ± 57.23^{X}	248.46 ± 15.77	180.27 ± 3.80^{X}			
	CP	209.13 ± 28.12^{X}	271.76 ± 15.76	212.91 ± 82.70^{X}			
	SA	$421.33 \pm 118.80^{Y,A}$	257.59 ± 66.76^{B}	$449.60 \pm 4.75^{Y,A}$	NS	***	**
Total PUFAₛ n-3	GR	45.17±2.56a	$68.47 \pm 38.54^{X,b}$	55.82 ± 14.00^{X}			
	DS	18.06 ± 2.87	18.29 ± 1.06^{Y}	14.20 ± 0.18^{Y}			
	CP	36.95 ± 3.11	46.40 ± 1.76	36.95 ± 15.19			
	SA	29.04 ± 6.86	15.41 ± 4.34^{Y}	24.76 ± 0.28	NS	***	NS
Total PUFAₛ n-6	GR	155.55 ± 9.69^{X}	230.17 ± 128.12	213.29 ± 57.07^{X}			
	DS	206.37 ± 53.91^{X}	229.33 ± 14.90	165.36 ± 3.94^{X}			
	CP	171.76 ± 24.92^{X}	224.72 ± 13.88	175.50 ± 67.28^{X}			
	SA	$442.71 \pm 72.16^{Y,A}$	240.98 ± 62.09^{B}	$423.07 \pm 4.93^{Y,A}$	NS	***	***

Values were expressed as mean ± standard errors. Abbreviations: ∑SFAₛ, sum of saturated fatty acids; ∑MUFAₛ, sum of monounsaturated fatty acids; ∑PUFAₛ, polyunsaturated fatty acids. LA: linoleic acid; ALA: α-linolenic acid; CLA: conjugated linoleic acid; AA: arachidonic acid; EPA: eicosapentaenoic fatty acids, DHA: docosahexaenoic fatty acids. Different lowercase (a,b = $p < 0.05$) or uppercase letters (A,B = $p < 0.01$) in the same row indicate significant differences among aging times (0, 3, and 7 days) within the same breed. Different lowercase (x,y = $p < 0.05$) or uppercase letters (X,Y = $p < 0.01$) in the same column indicate significant differences among breeds for the same aging time. On the right NS: not significant; (*) $p < 0.05$; (**) $p < 0.01$; (***) $p < 0.001$ indicate the effects of aging time, breed, and the time × breed interaction.

Table S3. Biological mean values (± standard errors) of color parameters (L*, a*, b*, chroma, and hue) in the *Longissimus thoracis et lumborum* (LTL) and *Quadriceps femoris* (QF) muscle cuts from the four goat breeds groups (GR, *Garganica*; DS, *Derivata di Siria*; CP, *Capra di Potenza*; SA, *Saanen*) during the aging period (n = 10 animals per group).

			Aging time			Effect		
**Items**	**Breed**	**Cut**	**T0 (0d)**	**T1 (3d)**	**T2 (7d)**	**T**	**B**	**T x B**
**L***	GR	LTL	52.27 ± 2.20^{X}	54.40 ± 1.56^{X}	53.58 ± 3.64^{X}			
		QF	52.30 ± 2.81^{X}	$53.87 \pm 2.39^{x,X}$	$53.14 \pm 1.73^{x,X}$			
			NS	NS	NS			
	DS	LTL	46.96 ± 1.09	48.90 ± 1.63	47.22 ± 0.76			
		QF	47.77 ± 1.97	52.43 ± 2.65^{x}	46.79 ± 0.87^{y}			
			NS	NS	NS			
	CP	LTL	$53.63 \pm 5.19^{A,X}$	50.09 ± 2.15	47.46 ± 0.93^{B}			
		QF	50.80 ± 2.17^{x}	48.28 ± 0.87^{y}	49.74 ± 1.02^{x}			
			*	NS	NS			
	SA	LTL	45.44 ± 2.65^{Y}	44.92 ± 2.43^{Y}	43.44 ± 2.06^{Y}			
		QF	$44.13 \pm 2.72^{y,Y}$	$44.12 \pm 2.61^{y,Y}$	$44.53 \pm 1.52^{y,Y}$			
			NS	NS	NS	NS	***	NS
**a***	GR	LTL	2.29 ± 1.44^{x}	$1.88 \pm 1.71^{x,X}$	1.91 ± 2.88			
		QF	2.06 ± 1.95^{X}	2.56 ± 1.33^{X}	3.02 ± 1.11			
			NS	NS	NS			
	DS	LTL	$2.74 \pm 0.53^{a,x}$	$5.19 \pm 0.74^{b,y}$	4.58 ± 0.43			
		QF	3.61 ± 0.80	2.83 ± 0.51^{X}	5.31 ± 0.48			
			NS	NS	NS			
	CP	LTL	4.65 ± 2.63	4.41 ± 1.04	3.72 ± 0.32			
		QF	3.38 ± 0.96	4.99 ± 0.88	4.41 ± 0.88			
			NS	NS	NS			
	SA	LTL	5.92 ± 2.31^{y}	5.85 ± 2.35^{Y}	5.87 ± 0.73			
		QF	6.64 ± 1.03^{Y}	7.04 ± 1.13^{Y}	6.31 ± 0.71			
			NS	NS	NS	NS	***	NS

**b***	GR	LTL	9.99 ± 0.49	8.69 ± 3.64	10.60 ± 1.10			
		QF	9.83 ± 2.09	8.95 ± 2.19	10.53 ± 1.23			
			NS	NS	NS			
	DS	LTL	11.74±1.24^{X}	8.63±1.05	8.61±0.79			
		QF	10.82±2.19	9.02±0.96	9.39±1.25			
			NS	NS	NS			
	CP	LTL	10.77±3.83	8.55±1.29	9.69±0.76			
		QF	10.13±3.45	12.45±4.93^{X}	10.56±0.91			
			NS	**	NS			
	SA	LTL	5.30 ± 3.94^{Y}	6.91 ± 1.27	8.13 ± 0.84			
		QF	8.60 ± 1.27	7.45 ± 1.03^{Y}	8.25 ± 1.43			
			NS	NS	NS	NS	***	NS
**Chroma**	GR	LTL	10.37 ± 0.36	9.32 ± 2.81	11.10 ± 1.01			
		QF	10.27 ± 1.68	9.48 ± 1.83	11.09 ± 8.07			
			NS	NS	NS			
	DS	LTL	12.10±1.25	10.17±1.08	9.85±0.69			
		QF	11.50±2.05	9.49±1.03	10.88±1.13			
			NS	NS	NS			
	CP	LTL	12.06±4.02	9.84±1.08	10.42±0.70			
		QF	11.45±3.54	13.54±4.81	11.52±1.13			
			NS	**	NS			
	SA	LTL	8.75 ± 2.35	9.14 ± 2.36	10.06 ± 0.96			
		QF	10.92 ± 1.35	10.28 ± 1.42	10.43 ± 1.46			
			NS	NS	NS	NS	NS	NS
**Hue**	GR	LTL	12.73 ± 8.34^{X}	7.05 ± 8.62^{y,X}	10.06 ± 14.92			
		QF	12.03 ± 13.90^{X}	18.08 ± 11.36^{X}	16.37 ± 8.07^{x}			
			NS	NS	NS			
	DS	LTL	13.59±2.36^{X}	31.60±3.48	27.88±2.94			
		QF	20.09±5.68^{x}	16.93±2.20^{X}	30.05±5.02			
			NS	NS	NS			
	CP	LTL	22.12±10.05^{x}	29.00±7.24^{x}	21.40±2.35			
		QF	22.09±7.48	24.05±3.71^{x}	22.34±3.93^{x}			
			NS	NS	NS			
	SA	LTL	35.24 ± 17.30^{y,Y}	39.66 ± 8.16^{Y}	35.92 ± 3.41			
		QF	37.53 ± 5.12^{y,Y}	43.55 ± 3.02^{y,Y}	38.02 ± 4.26^{y}			
			NS	NS	NS	NS	***	NS

Values are presented as mean ± standard errors. Different lowercase (a,b = $p < 0.05$) or uppercase letters (A,B = $p < 0.01$) in the same row indicate significant differences among aging time within the same muscle. Different lowercase (x,y = $p < 0.05$) or uppercase letters (X,Y = $p < 0.01$) in the same column indicate significant differences among breeds for the same muscle at a given aging time. Asterisks (*, **) indicate significant differences between LTL and QF within the same breed at a given aging time ($p < 0.05$ or $p < 0.01$, respectively), while NS denotes no significant difference. On the right NS: not significant; (***) p < 0.001 indicate the effects of aging time, breed, and the time × breed interaction.

Table S4. Biological mean values (± standard errors) of texture parameters in the *Longissimus thoracis et lumborum* (LTL) and *Quadriceps femoris* (QF) muscle cuts from the four goat breeds (GR, *Garganica*; DS, *Derivata di Siria*; CP, *Capra di Potenza*; SA, *Saanen*) during the aging period (n = 10 animals per group).

			Aging time			Effect		
**Items**	**Breed**	**Cut**	**T0 (0d)**	**T1 (3d)**	**T2 (7d)**	**T**	**B**	**T x B**
**Adhesiveness**	GR	LTL	-1.04 ± 1.04	-1.19 ± 1.30	-1.78 ± 0.64			
		QF	-1.6 ± 2.77^{a,X}	-2.35 ± 2.00^{b}	-1.01 ± 1.62			
			NS	NS	NS			
	DS	LTL	-1.50 ± 0.69^{Aa,X}	-1.79 ± 2.31^{B}	-1.59 ± 1.94^{b}			
		QF	-3.53 ± 2.83^{A,Y}	-1.24 ± 2.35^{B}	-2.64 ± 1.24^{Y}			
			**	NS	NS			
	CP	LTL	-2.41 ± 3.68^{Aa,X}	-1.56 ± 2.22^{B}	-1.00 ± 1.86^{b}			

		QF	-1.54 ± 3.38^{A,X}	-1.94 ± 2.60^{B}	-0.54 ± 1.26^{X}			
			NS	NS	NS			
	SA	LTL	-2.04 ± 0.56^{Y}	-0.60± 0.37	-1.77 ± 2.05			
		QF	-2.13 ± 0.59^{Y}	-2.97± 3.96^{A}	-0.61± 1.26^{B,X}			
			NS	**	NS	*	***	**
**Hardness, N**	GR	LTL	21.17 ± 1.76^{A,X}	16.75 ± 1.91^{B,X}	7.83 ± 3.80^{C,X}			
		QF	21.91 ± 1.01^{A,X}	11.03 ± 3.68^{B,X}	5.36 ± 4.36^{C,X}			
			**	**	**			
	DS	LTL	7.28 ± 2.90^{A,Y}	5.53 ± 2.27^{B,Y}	4.78 ± 2.02^{C,Y}			
		QF	5.24 ± 1.95^{A,Y}	4.63 ± 3.35^{B,Y}	3.75 ± 1.74^{C,Y}			
			**	**	**			
	CP	LTL	2.98 ± 1.88^{A,Z}	2.32 ± 1.64^{B,Z}	2.20 ± 2.23^{C,Z}			
		QF	18.96 ± 1.48^{A,Z}	14.13 ± 1.84^{B,Z}	20.43 ± 0.52^{C,Z}			
			**	**	**			
	SA	LTL	18.44 ± 2.02^{A,W}	15.22 ± 2.46^{B,W}	22.65 ± 0.29^{C,W}			
		QF	20.89 ± 1.12^{A,W}	18.35 ± 4.08^{B,W}	11.82 ± 4.60^{C,W}			
			**	**	**	***	***	***
**Springiness mm**	GR	LTL	0.96 ± 0.13^{X}	1.00 ± 0.00^{X}	0.99 ± 0.02			
		QF	1.03 ± 0.10^{X}	0.97 ± 0.03^{X}	1.00 ± 0.00			
			NS	NS	NS			
	DS	LTL	0.99 ± 0.00^{X}	1.00 ± 0.00^{X}	1.00 ± 0.00			
		QF	0.99 ± 0.03^{X}	1.00 ± 0.01^{X}	1.00 ± 0.00			
			NS	NS	NS			
	CP	LTL	1.00 ± 0.97^{X}	1.00 ± 0.00^{X}	0.99 ± 0.02			
		QF	1.00 ± 0.00^{X}	0.99 ± 0.02^{X}	0.99 ± 0.03			
			NS	NS	NS			
	SA	LTL	0.57 ± 0.30^{A,Y}	0.30 ± 0.02^{B,Y}	0.22 ± 0.03^{B}			
		QF	0.20 ± 0.02^{Y}	0.25 ± 0.03^{Y}	0.27± 0.03			
			**	NS	NS	*	***	***
**Gumminess, N**	GR	LTL	29.05 ± 1.92^{A,X}	21.39 ± 1.38^{B,X}	11.94 ± 6.90^{C,X}			
		QF	17.64 ± 7.52^{A,X}	16.44 ± 1.25^{B,X}	5.28 ± 3.72^{C,X}			
			**	**	**			
	DS	LTL	6.21 ± 2.89^{A,Y}	4.19 ± 3.83^{B,Y}	4.91 ± 2.04^{C,Y}			
		QF	4.02 ± 1.25^{A,Y}	4.99 ± 2.85^{B,Y}	6.64 ± 3.52^{C,Y}			
			**	**	**			
	CP	LTL	3.72 ± 1.93^{A,Z}	3.64 ± 1.03^{B,Z}	2.85 ± 2.73^{C,Z}			
		QF	20.59 ± 1.87^{A,Z}	18.74 ± 0.76^{B,Z}	16.52 ± 0.17^{C,Z}			
			**	**	**			
	SA	LTL	6.19± 3.68$^{A.W}$	6.23 ± 2.60$^{B.W}$	7.10 ± 3.18$^{C.W}$			
		QF	5.39 ± 0.44$^{A.W}$	5.25 ± 3.63$^{B.W}$	4.43 ± 3.36$^{C.W}$			
			**	**	**	***	***	***
**Chewiness, N × mm**	GR	LTL	31.53 ± 1.88^{A,X}	23.78 ± 1.61^{B,X}	12.22 ± 9.86^{C,X}			
		QF	22.37 ± 9.29^{A,X}	15.31 ± 2.80^{B,X}	5.38 ± 3.03^{C,X}			
			**	**	**			
	DS	LTL	8.57 ± 1.91^{A,Y}	7.12 ± 1.22^{B,Y}	8.44 ± 2.63^{C,Y}			
		QF	5.83 ± 1.66^{A,Y}	8.77 ± 3.37^{B,Y}	10.35 ± 2.92^{C,Y}			
			**	**	**			
	CP	LTL	4.53 ± 1.94^{A,Z}	4.23 ± 1.20^{B,Z}	3.31 ± 1.83^{C,Z}			
		QF	28.25 ± 1.87^{A,Z}	21.40 ± 1.80^{B,Z}	18.35 ± 0.49^{C,Z}			
			**	**	**			

	SA	LTL	$19.29 \pm 1.52^{A,W}$	$17.48 \pm 1.66^{B,W}$	$16.81 \pm 1.30^{C,W}$			
		QF	$15.31 \pm 1.66^{A,W}$	$13.76 \pm 1.18^{B,W}$	$11.34 \pm 0.68^{C,W}$			
			**	**	**	***	***	***
**Resilience**	GR	LTL	$0.24 \pm 0.12^{A,Yx}$	$0.10 \pm 0.03^{B,X}$	$0.14 \pm 0.05^{C,X}$			
		QF	0.13 ± 0.06	$0.14 \pm 0.15^{a,X}$	$0.11 \pm 0.03^{b,W}$			
			**	**	*			
	DS	LTL	0.22 ± 0.09^{Yy}	$0.19 \pm 0.07^{A,Y}$	$0.21 \pm 0.05^{B,Y}$			
		QF	0.13 ± 0.05^{a}	$0.14 \pm 0.03^{a,X}$	$0.10 \pm 0.04^{b,W}$			
			**	**	**			
	CP	LTL	$0.16 \pm 0.14^{A,X,Y}$	$0.09 \pm 0.06^{B.X}$	$0.28 \pm 0.11^{C.Z}$			
		QF	0.14 ± 0.06^{A}	0.17 ± 0.05^{A}	$0.21 \pm 0.04^{B.Y}$			
			*	**	**			
	SA	LTL	$0.18\pm 0.04^{A,YX}$	$0.18 \pm 0.04^{B,Y}$	$0.45 \pm 0.69^{C,W}$			
		QF	0.14 ± 0.07^{A}	$0.19 \pm 0.12^{Ba,Y}$	$0.15 \pm 0.04^{b,X}$			
			**	NS	**	***	***	***

Values are presented as mean ± standard errors. Different lowercase (a,b = $p < 0.05$) or uppercase letters (A,B = $p < 0.01$) in the same row indicate significant differences among storage times within the same muscle. Different lowercase (x,y = $p < 0.05$) or uppercase letters (X,Y = $p < 0.01$) in the same column indicate significant differences among breeds for the same muscle at a given storage time ($p < 0.05$ or $p < 0.01$, respectively). Asterisks (*, **) indicate significant differences between LTL and QF within the same breed at a given storage time ($p < 0.05$ or $p < 0.01$, respectively), while NS denotes no significant difference. On the right NS: not significant; (*) p < 0.05; (**) p < 0.01; (***) p < 0.001 indicate the effects of aging time, breed, and the time × breed interaction.

Figures

Figure S1. Changes of the main E-nose sensors in raw goat meat from *Longissimus thoracis et lumborum* (LTL) and *Quadriceps femoris* (QF) muscle cuts belonging to *Garganica* (GR), *Derivata di Siria* (DS), *Capra di Potenza* (CP), and *Saanen* (SA) breeds groups during the aging period (T0, 0 days; T1, 3 days; and T2, 7 days) (n = 10 animals per group).

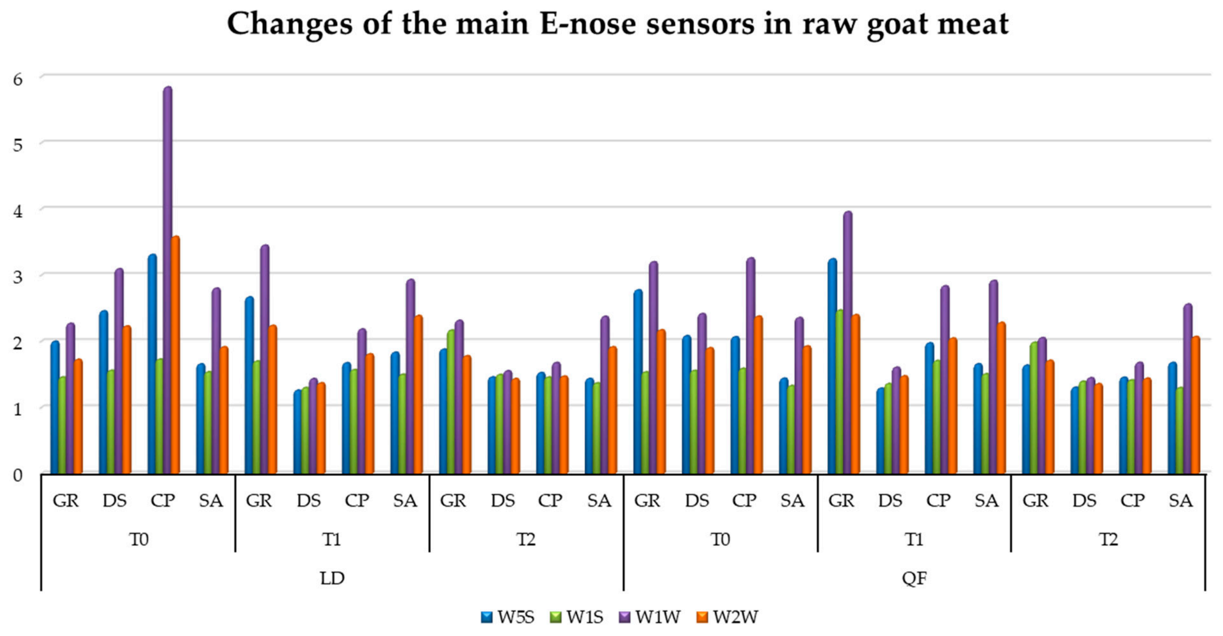

Sensor name: W5S, sensor very sensitive to nitrogen oxide; W1S, sensor very sensitive to methane; W1W, sensor very sensitive to sulphur compounds (H_2S); W2W, sensor sensitive to aromatic compounds and organic sulphur compounds.

Figure S2. Changes of the main E-nose sensors in cooked goat meat from *Longissimus thoracis et lumborum* (LTL) and *Quadriceps femoris* (QF) muscle cuts belonging to *Garganica* (GR), *Derivata di Siria* (DS), *Capra di Potenza* (CP), and *Saanen* (SA) breeds groups during the aging period (T0, 0 days; T1, 3 days; and T2, 7 days) (n = 10 animals per group).

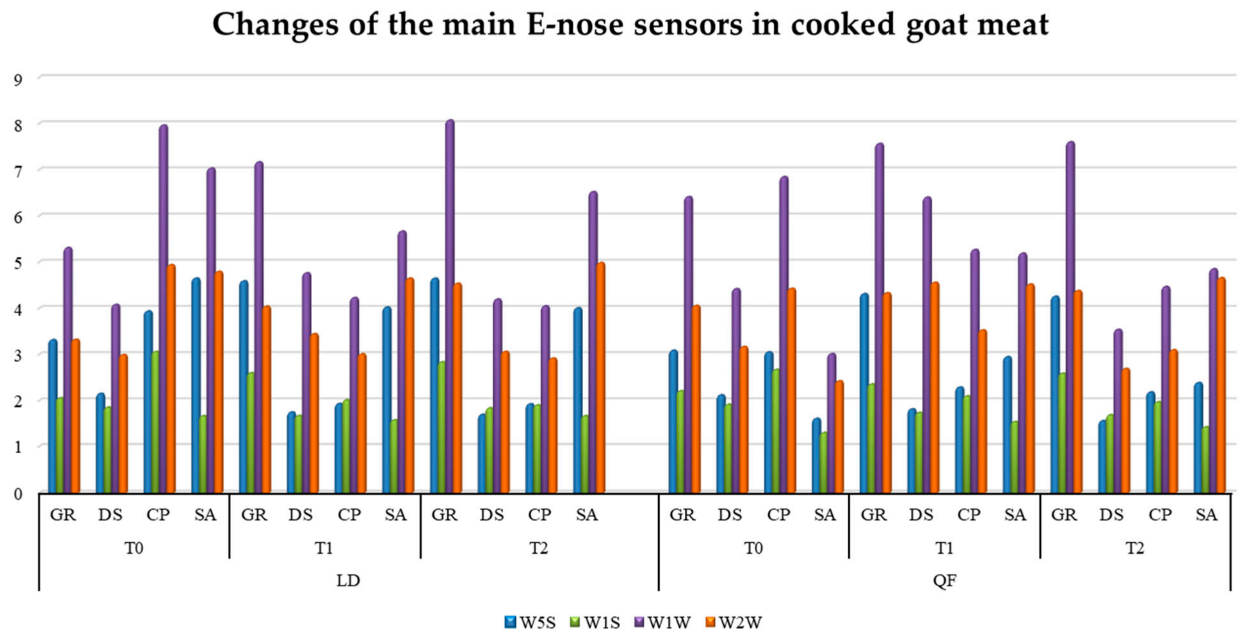

Sensor name: W5S, sensor very sensitive to nitrogen oxide; W1S, sensor very sensitive to methane; W1W, sensor very sensitive to sulphur compounds (H_2S); W2W, sensor sensitive to aromatic compounds and organic sulphur compounds.
